# Anti-angiogenic therapy in ovarian cancer: Current understandings and prospects of precision medicine

**DOI:** 10.3389/fphar.2023.1147717

**Published:** 2023-03-07

**Authors:** Chao Mei, Weijing Gong, Xu Wang, Yongning Lv, Yu Zhang, Sanlan Wu, Chunqi Zhu

**Affiliations:** ^1^ Department of Pharmacy, Union Hospital, Tongji Medical College, Huazhong University of Science and Technology, Wuhan, China; ^2^ Hubei Province Clinical Research Center for Precision Medicine for Critical Illness, Wuhan, China

**Keywords:** ovarian cancer, angiogenesis, biomarker, anti-angiogenic therapy, precision medicine

## Abstract

Ovarian cancer (OC) remains the most fatal disease of gynecologic malignant tumors. Angiogenesis refers to the development of new vessels from pre-existing ones, which is responsible for supplying nutrients and removing metabolic waste. Although not yet completely understood, tumor vascularization is orchestrated by multiple secreted factors and signaling pathways. The most central proangiogenic signal, vascular endothelial growth factor (VEGF)/VEGFR signaling, is also the primary target of initial clinical anti-angiogenic effort. However, the efficiency of therapy has so far been modest due to the low response rate and rapidly emerging acquiring resistance. This review focused on the current understanding of the in-depth mechanisms of tumor angiogenesis, together with the newest reports of clinical trial outcomes and resistance mechanism of anti-angiogenic agents in OC. We also emphatically summarized and analyzed previously reported biomarkers and predictive models to describe the prospect of precision therapy of anti-angiogenic drugs in OC.

## 1 Introduction

Ovarian cancer (OC) possesses the highest death rate among gynecological malignant tumors (Bray et al., 2018). While treatments have been improving over the past few decades, the survival rate has barely improved (Liu et al., 2021). According to statistics, 60%–80% of patients achieved complete remission after first-line therapy, but 80% of them finally die of therapy resistance or relapse (Agarwal and Kaye, 2003; Lengyel, 2010). Approximately 70% of patients relapse within 3 years after initial therapy (Viallard and Larrivee, 2017). Recurrent OC is incurable and the progression-free survival (PFS) decreases at each subsequent relapse treatment (Papa et al., 2016). The 5-year survival rate of OC patients is lower than 30%, while the PFS is about 16–22 months (Bray et al., 2018).

Angiogenesis is indispensable for tumor growth and development. Under physiological conditions, angiogenesis is a complicated and dynamic process that grows new vessels from existing ones, supplying the requirement alterations in tissue. However, angiogenesis is abnormally stimulated in the majority of cancers. Blood vessels provide oxygen and nutrients for tumors to survive and growth, without which tumors cannot develop to larger than 1–2 mm (Viallard and Larrivee, 2017). Therapeutic strategies targeting angiogenesis has been accepted for several types of solid tumors. The anti-angiogenic drug was the first targeted drug approved for OC. An increasing amount of innovative anti-angiogenesis agents are now being assessed in clinical trials of OC and mixed results are presented (Papa et al., 2016). However, individual differences and widespread resistance greatly limit the effectiveness of anti-angiogenic therapy. The above underscore the urgent need of discovering reliable molecular biomarkers to avoid resistance and improve the prognosis of OC patients.

## 2 Angiogenesis in tumor pathogenesis

In the pathological state of cancer, angiogenic signals will be exploited in a deregulated condition. Malignant cells release a series of growth factors, cytokines, and chemokines to stimulate quiescent cells to activate a cascade of signals. Except these, tumors may also trigger inflammatory reaction to recruit myeloid cells, releasing the stored soluble factors to facilitate the angiogenic response. These events quickly become deregulated and incline the balance toward secreting pro-angiogenic factors, thereby driving blood vessel growth (Ronca et al., 2015). These signals initiate formerly quiescent endothelial cell (EC) to sprout and proliferate on nearby vascular. Research indicated that tumor ECs lining blood vessels have a significant growth advantage, which probably divides 50 times quicker than in normal physiological conditions.

Normal vasculatures are arranged with a single-layer of tightly connected adherent ECs, which are polarized and aligned along the bloodstream for optimal perfusion. In comparison, tumor vasculature possesses the characteristics of abnormal structural dynamics, vascular immaturity, strikingly heterogeneous, tortuous, and high permeability (Dewhirst and Ashcraft, 2016; Dewhirst and Secomb, 2017; Zhang et al., 2019). Activated tumor ECs depolarize, slough off and piled up against each other, creating portals for malignant cells to entry the blood circulation. Tumor ECs are usually loosely connected and leaky, containing multiple fenestrations and trans-endothelial channels. In some tumors, these holes are more than 100 times larger than those in healthy blood vessels. Due to upregulated vessel resistance as well as disordered regulation, the bloodstream in the tumor is chaotic. The focal leaks and enhanced interstitial fluid pressure further create obstacles to the blood stream. The blood may flow rapidly in some vessels, but slowly in others, or even stagnant in some places (Carmeliet and Jain, 2011a). This pattern of blood flow leads to an abnormal microenvironment, seriously hindering the delivery of nutrients and drugs (Dewhirst et al., 1999). Fast-growing and metabolizing tumor cells constantly require abundant oxygen and nutrients. However, the non-productive blood vessel is far from the requirements of the tumor, which in turn stimulates tumor cells to produce an excess of pro-angiogenic factors. This leads to even more abnormal blood vessels, eventually creating an excess of the vicious cycle (Carmeliet and Jain, 2011a).

Tumor vessels often possess abnormal structure and function. This leads to a tumor microenvironment of hypoxia, inflammation, acidic pH and high interstitial hostile fluid pressure that interferes with the immune cellular function and the transport of chemotherapy drugs and oxygen. Therefore, abnormity of tumor vasculature leads to radiotherapy and chemotherapy resistance, and the escape of tumor cells through leaky vessels. In addition, hypoxia stimulates tumor and stromal cells to secrete large amounts of angiogenic factors, further exacerbating vascular disorders and accelerating non-productive angiogenesis in an interminable self-enhanced circle.

**FIGURE 1:**
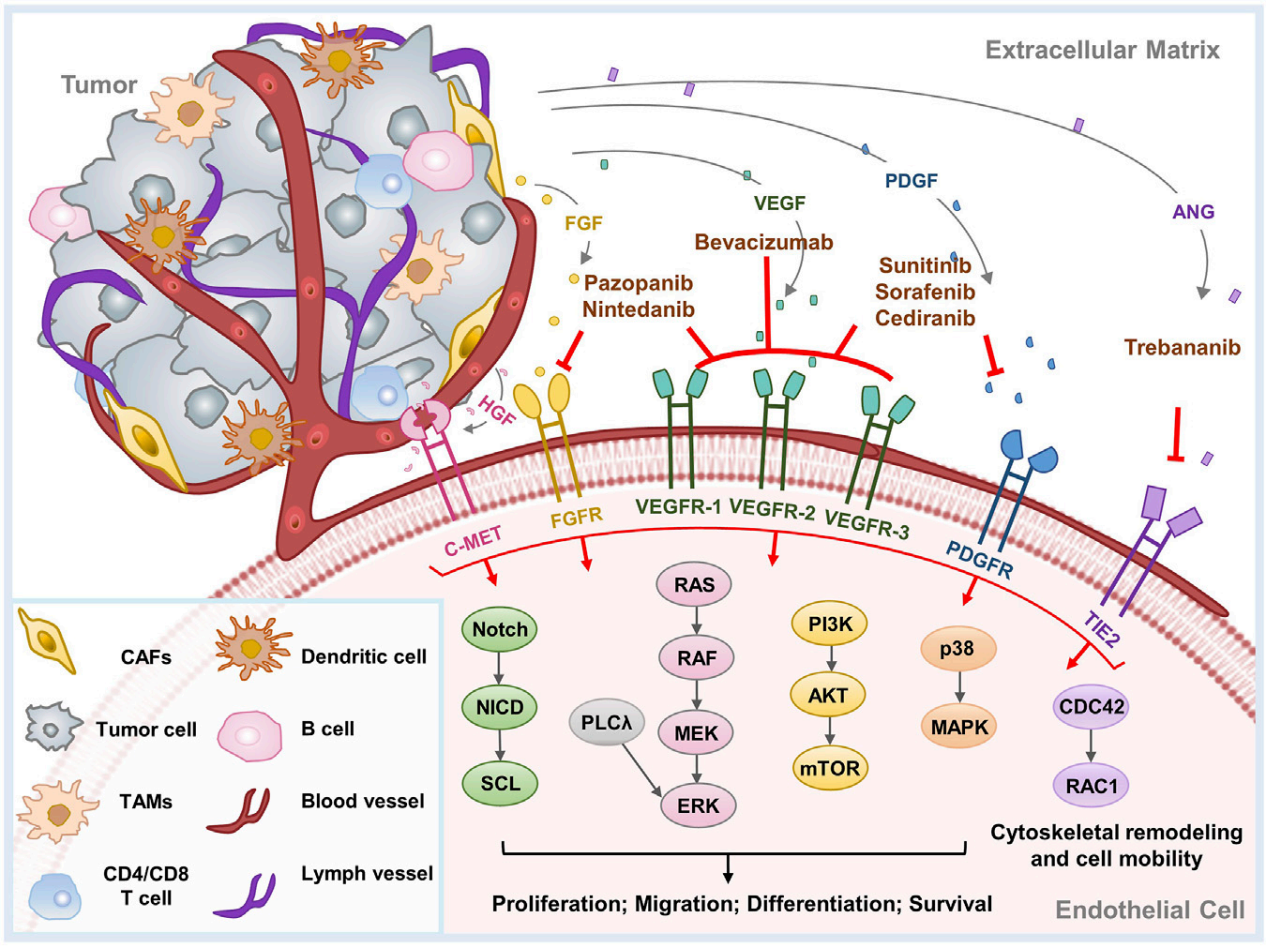
Major mechanisms of tumor angiogenesis and therapeutic agents implicated in OC. Tumor angiogenesis is induced by a series of proangiogenic factors. This diagram exhibits the principal angiogenic signaling pathways, as well as the molecular targets and therapeutic mechanisms of anti-angiogenic agents implicated in OC. CAFs, cancer associated fibroblasts; TAMs, tumor-associated macrophages.

To date, a large number of promoters of tumor angiogenesis have been discovered (Figure 1), such as the vascular endothelial growth factor (VEGF) family, angiopoietins (ANGPTs), fibroblast growth factors (FGFs), platelet-derived growth factor (PDGF), APLN (Apelin)/APLNR (G protein-coupled receptor APJ) pathway, hepatocyte growth factor (HGF)/hepatocyte growth factor receptor (c-MET), chemokines, Eph/Ephrin signaling, etc. Their targets, mechanisms, downstream signals and research status in OC are discussed below in detail.

## 3 Characteristics and functions of angiogenesis-related factors in OC

### 3.1 VEGF

VEGF, the most well-known pro-angiogenic factor, contains a group of ligands including VEGF-A to -D, as well as placental growth factor (PlGF) (Zhao and Adjei, 2015). VEGF can be secreted by malignant cells, fibroblasts, and inflammatory cells, usually in response to increased tissue hypoxia (Carmeliet and Jain, 2011b). VEGF binds to its receptor VEGFR tyrosine kinase and is activated to form homo- or heterodimers. VEGF-A tends to bind VEGFR-1 and 2. VEGF-B, PIGF-1, and PIGF-2 bind preferentially with VEGFR-1, while VEGF-C and -D mainly interact with VEGFR3 (Zhao and Adjei, 2015). The interaction between ligand and receptor triggers intracellular signaling cascades to promote the survival, proliferation, motility, permeability, and tube formation ability of ECs.

VEGF-A, VEGF-B, and PlGF play the uppermost functions in tumor angiogenesis, most of which are owing to the activation of VEGFR-2 by VEGF-A (Zhao and Adjei, 2015). PIGF binds to VEGFR-1 and its co-receptors neuropilin 1 (NRP1) and 2, which can directly facilitate vascular growth and maturation, or indirectly promote angiogenesis by recruiting monocyte-macrophage lineage cells and bone marrow-derived progenitors (De Falco, 2012). PIGF has been suggested as a potential participant in anti-VEGF resistance because of its upregulation in patients receiving anti-VEGF therapy (Willett et al., 2009; Bagley et al., 2011; Chiron et al., 2014). Aflibercept, which inhibits both VEGF-A and PIGF, has shown efficacy in cancer patient-derived xenograft models (Zhang and Lawler, 2007). VEGF-C and -D have the strongest binding affinity to VEGFR-3 and appear to be important in promoting lymph-angiogenesis.

The VEGF signaling is ubiquitous and upregulated in most cancer types. This overexpression is secondary to hypoxia and related transcription factors, like hypoxia-inducible factor -1α (HIF-1α) and HIF-2α. HIF-1α can stimulate several downstream proangiogenic growth factors, especially VEGF (Dewangan et al., 2019). Except this, insulin-like growth factor 1 (IGF-1), interleukin 6 (IL-6) (Salgado et al., 2002; Spiliotaki et al., 2011), and mutations in genes like p53, RAS, SRC, and VHL have also been shown to upregulate VEGF (White et al., 1997; Burger, 2011). Targeting VEGF can promote vascular normalization by recruiting pericytes, reducing the enlargement and tortuosity of vessels, and facilitating the normalization of the basement membrane (Carmeliet and Jain, 2011a). This results in a reduction in interstitial fluid pressure or edema, a transient increase in blood perfusion, oxygenation and improved efficiency of drug delivery.

In OC, VEGF signaling is highly activated and closely associated with metastatic potential, disease grade as well as poor prognosis (Wang et al., 2008). It is also a vital promotor of ascites production in the latter stage of OC cancer (Bamberger and Perrett, 2002; Numnum et al., 2006). VEGF activates its receptor VEGFR-2 on ECs to initiate multiple signaling pathways to mediate angiogenesis, for example, promoting EC proliferation and survival through extracellular signal-regulated kinase (ERK) and phosphatidylinositol 3-kinase (PI3K)/protein kinase B (AKT) pathways (Takahashi et al., 2001; Jiang and Liu, 2009); inducing cell invasion by activation of PI3K and Rho GTPases (Lamalice et al., 2007); mediating the basement membrane and extracellular matrix degradation as well as capillary sprout formation by mitochondrial membrane potential-2 (MMP-2), MMP-9, and urokinase plasminogen activator (uPA) (van Hinsbergh and Koolwijk, 2008; Jiang and Liu, 2009). VEGF–Akt–NF-κB signaling activation also induces an inflammatory response and promotes the recruitment of leukocytes, thereby contributing to the angiogenic process (Jiang and Liu, 2009). In addition, intracellular signaling including Janus kinase (JAK)-signal transducing activator of transcription (STAT), PI3K, and mitogen-activated protein kinase (MAPK) pathways have also been demonstrated to be related to VEGF signal (Banerjee and Kaye, 2011; Gavalas et al., 2013).

### 3.2 FGFs

The FGF family consists of 22 factors, 18 of which can bind and trigger the dimerization of their receptors FGFR1-4, initiating a series of intracellular signaling cascades (Turner and Grose, 2010). FGF is secreted by malignant cells, stromal cells, extracellular matrix and acts on ECs through paracrine signal. Among the FGF family, FGF1 and FGF2 exhibit uppermost proangiogenic abilities (Byron et al., 2010). In addition, the FGF/FGFR signal also contributed to tumor resistance to chemotherapy, radiotherapy, and targeted therapy (Katoh, 2016; Ghedini et al., 2018; Xie et al., 2020; Zhou et al., 2020). In OC, a spliced variant of FGFR and mutation events may confer binding sensitivity to the ligand and disrupt the downstream signaling cascade (Steele et al., 2001; Presta et al., 2005). The downstream signal pathways include the ERK/MAPK, JAK-STAT, phospsholipase-C (PLC)-inositol 1,4,5-triphosphate (IP3) cascade and PI3K-AKT pathway, which promotes angiogenesis, cell cycle progression as well as cell survival, proliferation and differentiation (Greenberg et al., 2008). FGFs also interferes with other signals like the Notch signal (Akai et al., 2005). In addition, FGF degrades the extracellular matrix *via* the promotion of plasminogen activators, MMPs, and collagenase (Turner and Grose, 2010). FGF also regulates cell metabolism through MYC-mediated glycolysis, which is essential for the proliferation, motility as well as sprouting of vascular ECs (Yu et al., 2017).

FGF signaling may be a compensatory angiogenesis mechanism that leads to VEGF-targeted therapy resistance. Increased FGF expression was found in patients with anti-VEGF therapy resistance. As FGF acts synergistically with VEGF to facilitate angiogenesis in cancer, simultaneously inhibiting the FGF signal effectively decreased vascular density and reverted sensitivity to anti-VEGF agents (Burbridge et al., 2013; Lee et al., 2015; Norden et al., 2015).

### 3.3 PDGF

There are four isoforms, PDGF-A to -D, in PDGF family (Heldin and Westermark, 1999). These ligands appear to have potent angiogenic activity by interacting with PDGFR-α and -β (Franco et al., 2011). PDGF signaling is involved in the survival, proliferation and migration of multiple types of cells (Ghedini et al., 2018). Hyperactivated PDGF signal, alone or accompanied with FGF and VEGF, result in excessive tumor angiogenesis, comprising but not limited to OC (Cao, 2013; Cantanhede and de Oliveira, 2017). In various types of cancer, aberrant PDGF signaling mediates the secretion of pro-angiogenic factors; promotion of pericyte recruitment and vascular maturation; facilitation of proliferation, migration, sprouting of ECs; interference with stroma formation; stimulation of lymph-angiogenesis and subsequent lymphatic metastasis (Levitzki, 2004; Cao, 2013; Zhao and Adjei, 2015). PDGF is also cross-linked to VEGF, by either converging their signaling cascades or being activated following the resistance to anti-VEGF therapy (Erber et al., 2004; Lu et al., 2008; Pietras et al., 2008). PDGF receptors are highly expressed in the pericytes of solid tumors, together with the critical role of PDGF signaling in mediating the immune microenvironment, targeting PDGF/PDGFR signal is expected to be a prospective therapeutic strategy (Heldin, 2013; Ostman, 2017; Bartoschek and Pietras, 2018; Papadopoulos and Lennartsson, 2018). The downstream signaling activated by the PDGF pathway includes PI3K/Akt, MAPK, Phospholipase C-γ (PLC-γ), Src, Ras and STAT, etc. (Gavalas et al., 2013).

According to previous studies, PDGF expression level in OC cells is approximately five to six-fold higher than that in normal ovarian ECs (Matei et al., 2002). In human OC tissue samples, PDGF was highly expressed in tumor stroma instead of the corresponding epithelial components, while PDGFR was mainly expressed in tumor stroma but not in OC cells (Li et al., 2022). In addition, high serum PDGF-BB and FGF2 were of prognostic significance. PDGFR-α and serum PDGF-BB expression have been reported to correlate with the prognosis of OC patients (Lassus et al., 2004; Madsen et al., 2012). Studies further supported the potency of PDGF in the anti-vascular therapeutic approach, by demonstrating that PDGFR blocking effectively improves the antitumor effect of bevacizumab (Lu et al., 2010). Taken together, PDGF is a key regulatory molecule in angiogenesis and ovarian carcinogenesis. Further studies are needed in the hope of developing more effective anti-tumor approaches.

### 3.4 ANGPTs

The ANGPTs family of ligands, ANGPT1 and ANGPT2, play a crucial role in vascular maintenance, remodeling, and development by interacting with the receptor tyrosine kinase TIE2 receptor (Aghajanian et al., 2012; Pujade-Lauraine et al., 2014; Coleman et al., 2017). ANGPT1 is an angiogenesis suppressor that mediates the neovascularization and maturation through Akt/surviving pathway, and is probably involved in the stabilization and protection of existing blood vessels (Thurston et al., 2000). As an endogenous antagonist of ANGPT1 function, ANGPT2 mainly mediates the remodeling process or vascular sprouting in response to VEGF (Scharpfenecker et al., 2005). Similar to cancer angiogenesis, ANGPT2 mostly promotes vascular instability and disruption that is characterized by unstable and leaky blood vessels (Tait and Jones, 2004; Reiss et al., 2009). ANGPT2 is involved in the predisposition of the endothelium towards the angiogenic statues required for angiogenic initiation and vascular destabilization (Scharpfenecker et al., 2005). It has also been suggested that ANGPT2 acts as an agonist in the absence of ANGPT1, while functioning as a dose-dependent antagonist when ANGPT1 exists (Yuan et al., 2009). The responders of ANGPT/Tie2 receptor include PI3K, MAPK/Erk, Ras signaling, etc. (Gavalas et al., 2013).

The serum levels of ANGPT1 and ANGPT2 were higher in ovarian tumor than normal ovaries, benign and/or borderline ovarian neoplasms (Sallinen et al., 2010; Sallinen et al., 2014). ANGPT1, ANGPT2 and ANGPT4 are upregulated in OC cells and tissues and indicate poor survival and a more aggressive phenotype, suggesting an attractive target in OC therapy (Brunckhorst et al., 2014). Upregulation of ANGPT2 is associated with decreased patient survival and resistance to anti-VEGF agents (Chae et al., 2010; Brunckhorst et al., 2014). Dual blocking of ANGPT2 and VEGFR2 effectively impaired glioma progression, promoted vascular normalization, blocked macrophage recruitment, and prolongered the prognosis of tumor-bearing mouse models (Kloepper et al., 2016; Peterson et al., 2016). This co-targeting effect has also been demonstrated in early colorectal, breast, and kidney cancer (Kloepper et al., 2016; Tuppurainen et al., 2017). However, ANG2/TIE2-induced tumor vessel instability may also make the established vasculature more resistant to anti-angiogenic agents (Gerald et al., 2013). Focusing on ANG/TIE2 signal to develop a targeted agent has proved to be challenging.

### 3.5 APLN/APLNR

APLN is a small, secreted peptide ligand of APLNR, which is predominantly expressed in ECs. APLN/APLNR signal is upregulated in several types of malignant T-cells and tumor ECs (Kalin et al., 2007; Seaman et al., 2007; Berta et al., 2010; Tolkach et al., 2019). APLN/APLNR signaling has been demonstrated to associate with neovascularization, tumor vessel density, microvascular proliferation, and tumor growth in other types of tumors (Sorli et al., 2006; Kalin et al., 2007; Sorli et al., 2007; Berta et al., 2010; Wu et al., 2017). APLN level is correlated with disease progress and worse clinical outcome, but its role in OC angiogenesis has seldom been identified (Berta et al., 2010; Heo et al., 2012; Lacquaniti et al., 2015; Feng et al., 2016). In OC, APLN functions as a mitogenic factor to promote cell proliferation (Hoffmann et al., 2017). APLN/APLNR signaling also drives OC metastasis in an angiogenesis-independent manner. Adipocyte-derived APLN promotes the uptake and utilization of lipids of OC cells, thus providing energy for the survival of OC cells in metastasis tissue (Dogra et al., 2021). Targeting APLN/APLNR for OC therapy is of certain prospect, but extensive research is still needed.

### 3.6 HGF/c-MET

HGF/c-MET exerts pro-angiogenic effects by both directly activating epithelial cells as well as indirectly stimulating VEGF and other proangiogenic factors (Cloughesy et al., 2017; Lopes-Coelho et al., 2021). c-MET is upregulated in patients with bevacizumab resistance (Shojaei et al., 2010). Concurrent administration of sunitinib (VEGFR and PDGFR receptor tyrosine kinases inhibitor (RTKI)) and HGF/c-MET inhibitors effectively inhibited angiogenesis and tumor growth (Lu et al., 2012). However, the combination of obinutuzumab (anti-c-Met) and bevacizumab has not brought significant clinical benefit (Rini et al., 2008; Kim et al., 2021).

c-MET is a prognostic factor of OC patients, targeting c-MET inhibits peritoneal dissemination, tumor invasion, and metastasis *in vivo* (Sawada et al., 2007; Mitra et al., 2011). Cabozantinib is the only approved TKI targeting VEGFRs, MET, and AXL (Maroto et al., 2022). A phase II trial reported the clinical benifit (objective response rate, 21%) and improved PFS (5.9 vs. 1.4 months) of cabozantinib in OC patients compared with the placebo arm (Vergote et al., 2017).

### 3.7 Eph/Ephrin signaling

The large family of receptor tyrosine kinases (RTK), Ephs and their binding ligands Ephrins exhibit oncogenic transformation, angiogenesis, vascular remodeling, malignant T-cell survival, migration, and invasion (Lisle et al., 2013). Ephs and Ephrins are sorted into two groups, A and B: EphrinA1-5, EphrinB1-3 and EphA1-10, EphB1-6. EphA2 and EphrinA1 expression is critical for tumor neovascularization and progression (Ogawa et al., 2000; Brantley et al., 2002; Cheng et al., 2003; Dobrzanski et al., 2004). Ephrb4-Ephrinb2 signaling was correlated with angiogenesis, tumor progression and anti-angiogenic drug resistance (Noren et al., 2004; Krusche et al., 2016; Uhl et al., 2018). The relationship between Ephrin and VEGF signaling has also been demonstrated. Ephrin-B2 regulates VEGF signaling by inducing the internalization of VEGFR2 and VEGFR3, thus mediating angiogenesis and lymphangiogenesis in both physiological and tumor conditions (Sawamiphak et al., 2010; Wang et al., 2010).

The expression of EphA1-2, B1-2, B2-4, -A1, -A5 was increased in OC cells (Herath et al., 2006; Alam et al., 2008). Ephrin-A1, -A5, and -A2 were associated with poor prognosis (Han et al., 2005; Herath et al., 2006). Ephrin-B2 and -B4 were in proportion to the disease stage (Alam et al., 2008). Ephrin-A4 is upregulated in OC and recognized as a novel tumor-initiating marker. PF-06647263, a monoclonal antibody against Ephrin-A4 conjugated with the DNA damage agent calicheamicin, showing limited antitumor efficiency in OC (Garrido-Laguna et al., 2019). EphA8 mRNA levels are upregulated in OC tissues compared with normal ovarian and fallopian tube tissues (Liu et al., 2016). High EphA8 protein level was correlated with later-stage, metastatic disease, serum levels of tumor and positive ascetic fluid, and has been regarded as a prognostic biomarker in epithelial ovarian cancer (EOC) patients (Liu et al., 2016). The above studies suggested the significant role of Eph/Ephrin signaling in OC.

### 3.8 Galectins

Apart from the factors described above, there are still several pro-angiogenic factors contribute to angiogenesis in OC. Galectins are a class of endogenous lectins, whose family members have been reported to correlate with cancer stage and disease recurrence of OC patients, as well as the proliferation, migration, invasion of OC cells (Shimada et al., 2020; Mielczarek-Palacz et al., 2022). Among them, Galectin-1 was the first identified and the most intensively studied member, which is an important proangiogenic factor in several types of carcinomas. Research has shown the positive correlation between Galectin-1 expression and number of micro vessels (Pranjol et al., 2019). Galectin-1 mediates angiogenesis mainly by enhancing the VEGF signaling pathway. Galectin-1 interacts with NRP-1, the co-receptor for VEGF, thereby activating VEGFR2 and downstream SAPK/JNK signaling to induce endothelial cell migration and adhesion. It has been shown that Galectin-1 can directly bind and activate VEGFR2, leading to anti-VEGF therapeutic resistance in the absence of VEGF. In addition to VEGF-VEGFR pathway, Galectin-1 also regulates H-Ras and Raf/MEK/ERK signals to promote endothelial cell activation, proliferation, migration and angiogenesis process (Martinez-Bosch and Navarro, 2020). As for the other member, Galectin-3 promotes angiogenesis *via* VEGF, basic FGF (bFGF) and modifies N-glycans on integrin αvβ3. Galectin-8 is expressed on the vascular endothelial cells of both normal and tumor-associated vessels, and facilities angiogenesis by promoting endothelial cell migration (Delgado et al., 2011; Troncoso et al., 2014).

### 3.9 Anti-angiogenic factors

Except the pro-angiogenic factors described above, anti-angiogenic factors, such as Thrombospondin-1 (TSP-1), Angioarrestin and Endostatin also play indispensable roles in OC progression and clinical treatment. TSP-1, the first identified endogenous anti-angiogenic factor, possesses a well-established anti-angiogenic and anti-tumor activity. TSP-1 is highly expressed in ovarian tumors. It can be secreted by a series of cell types including ECs, fibroblasts and immune cells, etc., and is highly located in the tumor stroma instead of tumor cells (Zhao et al., 2018). Based on its anti-angiogenic properties, high TSP-1 expression has been demonstrated to correlate with higher survival rates in OC, colon cancer, lung cancer and cervical cancer, etc. However, this conclusion is inconsistent or even opposite in other types of tumors, such as hepatocellular carcinoma, breast cancer and melanoma, etc. (Grossfeld et al., 1997; Zhao et al., 2018). These inconsistent conclusions led to controversy over its use as a survival predictor in different types of cancer. Similarly, existing studies has not shown a clear correlation between VEGF and TSP-1 expression in different tumor types. A recent meta-analysis included 24 studies revealed high TSP-1 expression may be a promising biomarker of poor prognosis in cancer, especially in breast and gynecologic cancers (Sun et al., 2020). ABT-510, a TSP-1 mimetic peptide, is the first TSP-1 inhibitor. ABT-510 effectively reduced the abnormal vasculature increased mature blood vessels within tumor, but failed to pass the phase II clinical study (Campbell et al., 2010; Zhao et al., 2018). Besides, the interaction between TSP-1 with CD47 directly inhibits tumor adaptive immunity. TAX2 is a selective antagonist against the interaction between TSP-1 and CD47. It effectively suppresses CD47 activation by targeting TSP-1, and reprograms highly vascularized ovarian tumors into poorly angiogenic ones, while concurrently activating anti-tumor immunity (Jeanne et al., 2021). TSP-1 derived peptides and peptide mimetics showed satisfied efficiency in the treatment of tumors driven by excessive angiogenesis, and hold great promise to become innovative drugs in the future.

Angioarrestin is another angiogenesis-inhibiting protein that endogenously produced by the tumor. Angioarrestin is downregulated in many types of tumor tissues and exhibited strong anti-angiogenic ability both *in vitro* and *in vivo* (Dhanabal et al., 2005). Angioarrestin is involved in the migration, adhesion and tube formation abilities of endothelial cells. Mechanistically, it has been reported to inhibit VEGF/bFGF-induced endothelial cell proliferation in a dose-dependent manner (Dhanabal et al., 2005). Endostatin is also an anti-angiogenic factor and has a potent activity on the migration, survival, proliferation and apoptosis of endothelial cells (Poluzzi et al., 2016). A genome-wide expression profiling demonstrated that about 12% of human genes are regulated by Endostatin in human endothelial cells (Abdollahi et al., 2004). Research indicated that Endostatin participates in MMPs, FAK/Ras/p38-MAPK/ERK, HIF-1α/VEGFA and Wnt signal (Dhanabal et al., 2005). Elevated Endostatin serum level may be a prognostic indicator for EOC patients. Either RGD-P125A-Endostatin-Fc fusion proteins alone or in combination with bevacizumab can effectively inhibit angiogenesis and OC progression (Jing et al., 2011).

## 4 Molecular targets and agents against angiogenesis

Bevacizumab has been approved in stage III or IV EOC patients after primary surgical resection, for either combining with carboplatin and paclitaxel, or maintaining as monotherapy (Table 1). In addition to bevacizumab, several other anti-angiogenic agents have also been tested clinical studies in OC (Table 2).

**TABLE 1:** Summary of anti-angiogenic agents in OC.

Drug name	Targets	Approved indications	Adaptation in OC	Route of administration
Bevacizumab	VEGFR	OC; Colorectal cancer; Non-small cell lung cancer; Recurrent glioblastoma; Hepatocellular carcinoma; Cervical cancer; Renal carcinoma; Breast cancer	Combination with chemotherapy; Maintenance monotherapy	I.V.
Pazopanib	VEGFR, PDGFR, FGFR, c-Kit, c-Fms	Soft tissue sarcoma; Advanced renal carcinoma	Clinical study	Oral
Nintedanib	VEGFR, FGFR, PDGFR	Idiopathic pulmonary fibrosis	Clinical study	Oral
Cediranib	VEGFR	/	Clinical study	Oral
Sunitinib	PDGFR, VEGFR, Flt3, c-Kit	Kidney cancer; Gastrointestinal stromal tumor; Neuroendocrine tumor	Clinical study	Oral
Sorafenib	VEGFR, PDGFR, Raf, ERK	Renal cell carcinoma, Hepatocellular carcinoma, Thyroid carcinoma	Clinical study	Oral
Trebananib	Tie2	/	Clinical study	I.V.

I.V., intravenous injection; OC, ovarian cancer.

**TABLE 2:** Summary of phase III studies of antiangiogenic agents in OC.

Clinical trials	Disease condition	Patient number	Drug	Treatment arm	Clinical outcomes		References
					PFS	OS	
OVAR 12	Newly diagnosed advanced OC	1366	Nintedanib	CBP + PTX + PBO	16.6	/	du Bois et al. (2016)
				CBP + PTX + Nintedanib	17.2 (HR, 0.84; 95% CI, 0.72 to 0.98]; *p* = 0.024)	/	
OVAR 16	Advanced OC	940	Pazopanib	PBO	12.3	/	du Bois et al. (2014)
				Pazopanib	17.9 (HR, 0.77; 95% CI, 0.64 to 0.91; *p* = 0.0021)	/	
ICON6	PT-sensitive recurrent OC	456	Cediranib	PBO + CBP, PBO maintenance	8.7	19.9	Ledermann et al. (2016), Ledermann et al. (2021)
				Cediranib + CBP, PBO maintenance	10.1 (HR, 0.67; 95%CI, 0.53-0.87; *p* = 0.0022)	/	
				Cediranib + CBP, Cediranib maintenance	11.1 (HR 0.57, 0.44-0.72, *p* < 0.00001)	27.3(HR, 0.85; 95% CI, 0.66-1.10; *p* = 0.21)	
NRG-GY004	Recurrent Pt-sensitive OC	565	Cediranib	chemotherapy	10.3	/	Liu et al. (2022)
				Olaparib	8.2 (HR, 1.2; 95%CI, 0.93-1.5)	/	
				Olaparib + Cediranib	10.4 (HR, 0.856; 95%CI, 0.66-1.10; *p* = 0.077)	/	
TRINOVA-1	Recurrent OC	919	Trebananib	Weekly PTX + PBO	5.4	18.3	Monk et al. (2016b), Vergote et al. (2019a)
				Weekly PTX + Trebananib	7.2 (HR, 0.66; 95%CI, 0.57-0.77; *p* < 0.0001)	19.3 (HR, 0.95; 95%CI, 0.81-1.11; *p* = 0.52)	
TRINOVA-2	Recurrent OC	223	Trebananib	PLD + PBO	7.2	17	Monk et al. (2014)
				PLD + Trebananib	7.6 (HR, 0.92; 95%CI, 0.68-1.24; *p* = 0.57)	19.4(HR, 0.94; 95%CI, 0.64-1.39; *p* = 0.76)	
TRINOVA-3	Advanced OC	1015	Trebananib	PBO + PTX + CBP	15.0	43.6	Marth et al. (2017)
				Trebananib + PTX + CBP	15.9 (HR, 0.93; 95%CI, 0.79-1.09; *p* = 0.36)	46.6(HR, 0.99; 95%CI, 0.79-1.25; *p* = 0.94)	
AGO-OVAR16	Stage II-IV EOC	940	Pazopanib	PBO maintenance	17.9	18.3	du Bois et al. (2014), Vergote et al. (2019b)
				Pazopanib maintenance	12.3 (HR, 0.77; 95%CI, 0.64-0.91; *p* = 0.0021)	59.1 (HR, 0.960; 95% CI: 0.805-1.145; *p* = 0.6431)	

OC, ovarian cancer; CBP, carboplatin; PTX, paclitaxel; PBO, placebo; HR, hazard ratio; CI, confidence interval; PLD, pegylated liposomal doxorubicin.

### 4.1 Bevacizumab

Bevacizumab (Avastin^®^) is a humanized anti-VEGF monoclonal antibody. It was the first target medicine approved in 2014 and used for platinum-resistant OC in combination with chemotherapy (Monk et al., 2016a). It exerts therapeutic efficiency by blocking VEGF-A to bind VEGFR, destroying existing vessels, disturbing neovascularization, and releasing intratumor pressure, etc. (Reinthaller, 2016). Studies have shown that blocking VEGF signaling not only leads to the depletion of tumor vascularization, but also promotes the normalization of the remaining blood vessels in morphology and function. In addition, the pericyte coverage of remaining vessels increased to about 75% after bevacizumab treatment, compared with 7% in the placebo group (Arjaans et al., 2013).

The application of bevacizumab in OC was initially used as monotherapy in pretreated patients. The GOG-0170D trial evaluated the benefit of bevacizumab single agent in 62 recurrent OC patients that had been treated with up to two prior lines of chemotherapy. Bevacizumab was well tolerated. The ORR was 21%. PFS and overall survival (OS) was 4.7 and 17 months respectively (Burger et al., 2007). Other phase II studies evaluated the benefit of bevacizumab in OC patients that had experienced disease progression after multiple chemotherapeutic regimens (Monk et al., 2006; Cannistra et al., 2007). Single-agent bevacizumab showed modest benefits, but less than combination therapy (Fuh et al., 2015).

In 2011, the outcomes of two prominent phase III trials, ICON7 and GOG-0218, were published simultaneously, which were the first attempt to add bevacizumab to standard adjuvant chemotherapy as a frontline maintenance of OC. In GOG-0218, incorporation of bevacizumab within 10 months after carboplatin (CBP) and paclitaxel (TAXOL) chemotherapy has been shown to prolong the PFS for approximately 4 months in 1873 newly diagnosed advanced EOC patients (medium PFS, 14.1 vs. 10.3 months; 95% CI, 0.625-0.824; *p* < 0.001) (Burger et al., 2011). As for the ICON7 trial, bevacizumab combination therapy improved the PFS to 24.1 months in 1528 OC patients compared with CBP and TAXOL chemotherapy alone (22.4 months). The benefit was more obvious in patients with high progression risk (PFS, 18.1 vs. 14.5 months; OS, 36.6 vs. 28.2 months) (Perren et al., 2011).

Platinum (Pt) resistance is a serious problem that hinders the therapeutic benefit of OC. Factors leading to Pt resistance are various, including angiogenesis, hypoxia, immune infiltration, and abnormal regulation of breast cancer susceptibility gene (BRCA), ATP binding cassette subfamily B member 1 (ABCB1) and cyclin E1 (CCNE1), etc. (Pennington and Swisher, 2012; Patch et al., 2015). Anti-angiogenic drugs exert a satisfying therapeutic benefit in Pt-resistant OC (Haunschild and Tewari, 2020). An open-label, randomized, phase III trial, AURELIA, demonstrated that bevacizumab incorporated with standard-of-care chemotherapy (TAXOL or topotecan (TPT) or pegylated liposomal doxorubicin (PLD)) improved the PFS of Pt-resistant OC patients compared to chemotherapy alone (medium PFS, 6.7 vs. 3.4 months; HR, 0.42; 95%CI, 0.32-0.53) (Pujade-Lauraine et al., 2012). The subsequent analysis indicated combining with TAXOL was the most effective regimen (Poveda et al., 2015). Based on the AURELIA trial, the Food and Drug Administration (FDA) had approved bevacizumab plus weekly TAXOL, PLD, or TPT for patients with Pt-resistant OC (Pujade-Lauraine et al., 2014).

Bevacizumab combination therapy has also been evaluated in Pt-sensitive OC patients. A phase III trial, OCEANS, was performed in 484 patients with Pt-sensitive recurrent OC. The medium PFS was 12.4 months in the bevacizumab/gemcitabine/CBP and 8.4 months in chemotherapy only group (HR, 0.48; 95% CI, 0.39-0.61) (Aghajanian et al., 2012). GOG-0213 trail evaluated the efficiency of combining bevacizumab with CBP and TAXOL. The median OS (49.6 vs. 37.3 months; HR, 0.823; 95% CI, 0.680-0.996; *p* = 0.0447) was improved in the bevacizumab group compared with chemotherapy only group (Coleman et al., 2017). Both therapy regimens in the above two trials have been approved by FDA for this usage. The MITO16b phase III trial was performed in 406 Pt-sensitive recurrent OC patients and compared the PFS benefits of bevacizumab combination with standard chemotherapy. Continuing bevacizumab combination therapy significantly prolonged the PFS (medium PFS, 11.8 vs. 8.8 months; HR, 0.51; 95% CI, 0.41-0.65; *p* < 0.0001) (Pignata et al., 2021).

Taken together, the vast majority of clinical studies suggested that bevacizumab significantly extended PFS in OC patients by several months, while the improvement in OS was not obvious. Up to now, mechanism studies focused on bevacizumab resistance have achieved certain progress, and several multitargeted antiangiogenic agents have been tested in clinical studies. However, no effective clinical methods has been applied to overcome bevacizumab resistance. In addition, there is growing evidence that the combination of bevacizumab with immunotherapy or PARP inhibitors may improve the therapeutic outcome of OC patients. Further attempts of novel combination therapies hold promising prospects and are one of the major trends in antiangiogenic therapy.

### 4.2 Pazopanib

Pazopanib, an oral tyrosine kinase inhibitor (TKI) of multiple targets, inhibits VEGFR, PDGFR-α and -β, FGFR-1 and -3 and c-Kit. Pazopanib treatment significantly reduced the tumor microvessel density and pericyte coverage in the mouse orthotopic OC model (Merritt et al., 2010). Pazopanib has been approved by the FDA and European Medicines Agency (EMA) for soft tissue sarcoma as well as advanced renal carcinoma therapy. Although not yet approved in OC, many phase II and III clinical trials have evaluated the potential role of pazopanib in the therapy of OC (Plummer et al., 2013; du Bois et al., 2012; Davidson and Secord, 2014). The AGO-OVAR16 study assessed the potential role of pazopanib maintenance therapy in 940 OC patients without progressive disease after receiving the first-line chemotherapy. Pazopanib, when given as maintenance therapy, yielded a meaningful improvement in median PFS (17.9 v 12.3 months; HR, 0.77; 95% CI, 0.64-0.91; *p* = 0.0021), albeit with added adverse event-induced therapy interruption (33.3% vs. 5.6%)**.** However, no significant benefit of OS was identified (du Bois et al., 2014).

So far, there have been few phase III clinical trials of pazopanib in OC treatment, but it has already exhibited clear clinical benefit and future studies will gradually establish its value in OC. In addition, the curative effect of pazopanib in bevacizumab-resistant patients remains undefined and requires further investigation. Importantly, it is more necessary to discover valid predictive biomarkers to avoid potential toxicity and identify patients who are more likely to benefit from pazopanib treatment. A previous study showed that [^18^F] Fluciclatide-PET uptake parameters may predict clinical outcomes of pazopanib treatment in patients with platinum-resistant/refractory OC, but studies in larger sample size are still needed for validation (Sharma et al., 2020). Besides, soluble VEGFR-2 and IL-8 have been revealed to be potential predict biomarkers in predict the therapeutic efficiency of pazopanib (Davidson and Secord, 2014). In summary, though the application of pazopanib in OC is still being explored and debated, the results of combination studies and further phase III studies will hopefully provide a rational foundation for the optimal role of pazopanib in OC treatment.

### 4.3 Nintedanib

Nintedanib (BIBF 1120) is an orally available, multitargeted antiangiogenic agents that approved for idiopathic pulmonary fibrosis treatment by FDA in 2014. Nintedanib competitively inhibits RTK (including VEGFR, FGFR, PDGFR-α and -β and FLT3 kinases) as well as non-RTK (including lymphocyte-specific protein tyrosine kinase (Lck), tyrosine-protein kinase Lyn (Lyn) and proto-oncogene tyrosine-protein kinase Src (Src)) (Cortez et al., 2018). Dynamic MRI assessments indicated that nintedanib treatment led to significant reduction of blood flow in about 55% OC patients. It also promotes the vascular normalization and regression of tumor in pre-clinical models (Khalique and Banerjee, 2017). A phase II trial investigated the efficacy of nintedanib maintenance therapy after chemotherapy for relapsed OC. 83 patients were included in this study. 36 weeks PFS rate was improved to some extent, but no statistical significance (16.3% and 5.0%; HR, 0.65; 95% CI, 0.42-1.02; *p* = 0.06) (Ledermann et al., 2011). A recent phase II study assessed the benefit and tolerance of combining nintedanib with oral cyclophosphamide in 117 relapsed OC. The median OS in nintedanib and placebo group were 6.8 and 6.4 months respectively (HR, 1.08; 95% CI, 0.72-1.62; *p* = 0.72), and the 6-month PFS rates were 29.6% and 22.8%, respectively (*p* = 0.57). No meaningful improvement was observed when nintedanib was added to oral cyclophosphamide (Hall et al., 2020). Another phase II trial investigated whether nintedanib is effective in bevacizumab-resistant recurrent EOC. According to research findings, nintedanib monotherapy was tolerable and showed minimal efficiency in bevacizumab-resistant EOC patients (Secord et al., 2019). In the AGO-OVAR 12 phase III clinical trial, nintedanib combined with CBP and TAXOL had a modest efficacy in patients with FIGO IIB-IV OC (PFS, 17.2 vs. 16.6 months; HR, 0.84; 95%CI, 0.72-0.98; *p* = 0.024), but was also accompanied by more gastrointestinal adverse events (Secord et al., 2019). The follow-up study continually reported no significant different in OS (62.0 vs. 62.8 months; HR, 0.99; 95% CI, 0.83-1.17; *p* = 0.86). The updated PFS difference was in line with the primary report (17.6 vs. 16.6 months; HR, 0.86; 95% CI, 0.75-0.98; *p* = 0.029) favoring nintedanib (Ray-Coquard et al., 2020).

Based on the limited prognostic benefit and non-negligible toxic effects reported in clinical trials to date, it is not expected to approve nintedanib for OC therapy. Nevertheless, these studies were informative and suggested the demand of patient selection and tolerated therapy. Nintedanib may have a role in recurrent OC. The ongoing clinical trials and predictive biomarker identification will help to determine this (Khalique and Banerjee, 2017).

### 4.4 Cediranib

Cediranib (AZD2171) is an oral TKI that inhibits VEGFR-1, VEGFR-2, VEGFR-3 and c-kit. In preclinical models of OC, cediranib treatment led to significantly reduction of tumor vascular density and vessel regression (Ruscito et al., 2016). A phase II trial reported a significant activity of cediranib in Pt-sensitive instead of Pt-resistant patients with recurrent OC (Hirte et al., 2015). The ICON6 phase III study further evaluated whether orally given cediranib plus Pt-based chemotherapy and continued as maintenance therapy provided PFS benefits in 456 Pt-sensitive OC patients. A significantly prolonged PFS was found in the cediranib combination and maintenance group (11.0 vs. 8.7 months; HR, 0.56; 95%CI, 0.44-0.72; *p* < 0.0001), accompanied by added toxic effects (Ledermann et al., 2016). However, no significant difference was found in the extended follow-up of OS results (OS, 27.3 vs. 19.9 months; HR, 0.86; 95% CI, 0.67-1.11; *p* = 0.24). Even so, the result of OS was underpowered due to several limitations like drug supply restriction and the non-proportionality of the survival curves, and further research should be undertaken (Ledermann et al., 2021).

Olaparib is a poly (ADP‐ribose) polymerase (PARP) inhibitor that applied for OC therapy, but widespread resistance greatly hindered its clinical benefit. Better strategies and potential combination administrations are in urgent need to overcome the resistance. A phase II study investigated whether combining cediranib with olaparib could improve the PFS of patients with Pt-sensitive recurrent OC. Median PFS were 9.0 and 17.7 months in the olaparib monotherapy and cediranib plus olaparib group, respectively (HR, 0.42; 95% CI, 0.23-0.76; *p* = 0.005) (Liu et al., 2014). The follow-up study characterized OS and updated PFS outcomes. The updated PFS result was consistant (16.5 vs. 8.2 months; HR, 0.50; *p* = 0.007). The OS showed no statistical difference (44.2 vs. 33.3 months, HR, 0.64; *p* = 0.11). Notably, for the subgroup of patients that did not carry deleterious germline BRCA1/2 mutation, both OS (37.8 vs. 23.0 months; *p* = 0.047) and PFS (23.7 vs. 5.7 months; *p* = 0.002) were significantly improved by adding cediranib to olaparib, suggesting that the further study should designed on the basis of BRCA status (Liu et al., 2019a). The EVOLVE trail evaluated the benefit of cediranib plus olaparib when confronted with PARPi treatment resistance. The cediranib–olaparib combination was tolerable and the efficiency was various in patients with different resistance mechanism. Individuals with upregulated ABCB1 and/or abnormal homologous recombination repair activity should probably be considered for other treatment options (Lheureux et al., 2020).

Several clinical trials have compared the clinical benefit of olaparib and/or cediranib with that of chemotherapy. A phase II study reported no PFS improvement was identified in cediranib plus olaparib *versus* chemotherapy in unscreened, heavily pretreated Pt-resistant OC patients (Colombo et al., 2022). Consistent findings were reported in NRG-GY004 phase III trial which performed in 565 recurrent Pt-sensitive OC patients. The median PFS were 10.3, 8.2, and 10.4 months in the chemotherapy, olaparib, and olaparib + cediranib groups, respectively. Combining olaparib with cediranib showed no more PFS benefit than chemotherapy (HR, 0.86; 95%CI, 0.66-1.10; *p* = 0.077). However, for the subgroup with germline BRCA mutation, significant clinical activity was observed both in olaparib alone or in combination with cediranib (Liu et al., 2022). The above studies suggested the critical role of valid genetic biomarkers in screening susceptible individuals and predicting the efficacy of cediranib.

In addition to the clinical trials described above, numerous studies are ongoing. A phase II trial aims to compare the benefit and tolerability of olaparib plus cediranib *versus* olaparib monotherapy in Pt-resistant OC (Mansouri et al., 2021). The ICON 9 phase III randomized study assessed the maintenance treatment of olaparib plus cediranib in relapsed Pt-sensitive OC. The trail is ongoing and the primary results are expected in 2024 (Elyashiv et al., 2021).

Although not yet approved by FDA, the landscape of cediranib in OC therapy appears promising. Cediranib exhibited encouraging results when combined with chemotherapy or olaparib. Nevertheless, many key questions remain to be addressed in the future, such as which clinical regimen provides the best benefit; biomarkers to identify patients with higher probability to benefit are urgently needed; the unclear role of cediranib in bevacizumab resistant patients. In the near future, the outcomes of phase II/III clinical trials will help to better establish the role of cediranib in OC treatment.

### 4.5 Sunitinib

Sunitinib is a multiple-target TKI that inhibits PDGFR, VEGFR, Flt3, and c-Kit. The FDA granted sunitinib for the treatment of advanced kidney cancer and partial gastrointestinal stromal and neuroendocrine tumors, while its application in OC remains in clinical trials (Leone Roberti Maggiore et al., 2013). In a xenograft mouse model, sunitinib therapy significantly reduced the tumor microvascular density, and also inhibited tumor growth and peritoneal metastasis (Bauerschlag et al., 2010). The AGO-OVAR2.11 phase II trial showed that sunitinib exhibited feasibly and moderate activity in patients with recurrent Pt-resistant OC, and the non-continuous therapy schedule showed better superiority compared with continuous treatment (Baumann et al., 2012). Attached to this, the predictive value of VEGF, VEGFR-3 and Ang-2 was evaluated. Decreased serum Ang-2 levels were found to associate with longer PFS (8.4 vs. 2.7 months). However, the difference is not significant (*p =* 0.0896) and further research is needed (Bauerschlag et al., 2013). Another phase II trial also reported a modest activity of 50 mg intermittent regimen of sunitinib monotherapy in recurrent Pt-sensitive OC (Biagi et al., 2011). The dosage regimen may be a vital consideration in further studies of sunitinib in OC (Biagi et al., 2011). Susana M Campos et al. demonstrated a modest response rate (8.3%) of sunitinib in recurrent OC in a phase II trial (Bodnar et al., 2011). Another phase II evaluation of sunitinib also reported limited effectiveness in persistent or recurrent clear cell OC (Campos et al., 2013).

Based on the above studies, sunitinib exhibited moderate antitumor activity together with acceptable toxicity in the OC treatment. However, given that serious adverse events have been reported (Abdollahi et al., 2004; Dhanabal et al., 2005; Poluzzi et al., 2016; Jeanne et al., 2021), more insight understanding of toxicity, elucidating the specific toxic mechanisms, and determination of optimal administration dosage are required in the future. It is also important to identify predictable biomarkers to guide individualized medication. In addition, current clinical studies have not attempted the combination therapy of sunitinib with cytotoxic agents, which may significantly improve therapeutic outcome and control toxicity.

### 4.6 Sorafenib

Sorafenib targets multiple kinases including VEGFR, PDGFR, Raf, MEK and ERK. It has been approved for renal cell carcinoma, hepatocellular carcinoma and differentiated thyroid carcinoma by FDA. A phase II trial indicated sorafenib provided no adequate objective response when given as a third-line therapy in EOC (Chan et al., 2018). In another phase II study, sorafenib was assessed as maintenance therapy in 246 EOC patients that had achieved a complete response in first-line therapy. Compared with placebo group, no obvious PFS improvement was achieved in sorafenib 400 mg BID treatment (median PFS, 12.7 vs. 15.7 months; HR, 1.09; 95% CI, 0.72-1.63). Adverse effects induced discontinuations were more frequently in the sorafenib group (37.4% vs. 6.5%) (Herzog et al., 2013).

A phase II study evaluated the efficiency and tolerability of sorafenib plus CBP/TAXOL in EOC. This study was terminated after patients occurred life-threatening toxicities (Erber et al., 2004), suggesting that sorafenib plus CBP/TAXOL cannot be recommend as neoadjuvant treatment in patients with primary advanced OC (Polcher et al., 2010). This result was consistent with another randomized phase II trial, which reported that the combination of sorafenib to standard TAXOL/CBP provided no benefit but more serious toxicity in patients with advanced EOC (Hainsworth et al., 2015). Another randomized phase II trial compared the benefit of sorafenib monotherapy, or combined with CBP/TAXOL in Pt-sensitive EOC. The median PFS of sorafenib monotherapy and combination group were 5.6 and 16.8 months, respectively (*p* = 0.012), while difference was not observed in OS (25.6 vs. 25.9 months, *p* = 0.974) (Schwandt et al., 2014).

The combination of sorafenib with TPT was evaluated in Pt-resistant or -refractory OC. Sorafenib combination significantly prolonged PFS *versus* placebo (6.7 vs. 4.4 months; HR, 0.60; 95% CI, 0.43-0.83; *p* = 0.0018) (Chekerov et al., 2018). However, another phase II trial reported conflicting results, pointing a significant toxicity but modest clinical efficacy in Pt-resistant OC patients (Ramasubbaiah et al., 2011). The combination of sorafenib with TPT still required further investigation.

Continuous daily sorafenib combined with bevacizumab caused moderate toxicity in OC patients, whereas intermittent sorafenib plus bevacizumab had promising clinical efficacy with few side effects (Lee et al., 2010). A phase II trial reported potential clinical activity of bevacizumab plus sorafenib in bevacizumab-naive, Pt-resistant OC, whereas no activity was observed in the bevacizumab-prior group (Lee et al., 2020).

According to previous phase II studies, sorafenib showed limited clinical benefit in advanced relapsing OC when given as single agent or combination therapy. Sorafenib in combination with cytotoxic agents also provided less benefit, and severe adverse events were reported. Nonetheless, sorafenib combined with bevacizumab exhibited encouraging efficacy in advanced OC patients, but the cumulative toxicity also posed an ongoing therapeutic challenge. Future research should therefore focus on developing reliable predictive biomarkers to guide patient selection, optimal combination, order and dose of administration, so as to maximize clinical benefit and minimizing toxicity.

### 4.7 Trebananib

Trebananib (ANG386) targets and blocks the binding of ANGPT to their receptor Tie2. A study used photoacoustic tomography to detect changes in tumor vascularization in response to trebananib treatment. It showed that trebananib induced obvious vessel regression and reduced vessel density. It is worth noting that trebananib treatment did not completely block angiogenesis but promoted more stable and less permeable residual vascular structures (Bohndiek et al., 2015). The TRINOVA-1 trial assessed the benefit of trebananib plus TAXOL in 919 recurrent EOC patients. The median PFS was meaningfully improved in the trebananib plus TAXOL arm compared with placebo arm (7.2 vs. 5.4 months; HR, 0.66; 95% CI, 0.57-0.77; *p* < 0.0001). The adverse events were 125 (28%) and 159 (34%) in the placebo monotherapy group and trebananib combination group, respectively (Monk et al., 2014). The ENGOT-ov-6/TRINOVA-2 study investigated the potential benefit of combining trebananib with PLD in 223 recurrent EOC patients. The objective response rate (ORR, 46% vs. 21%) and duration of response (DOR, 7.4 vs. 3.9 months) were improved, while the median PFS had no obvious improvement (7.6 vs. 7.2 months; HR, 0.92; 95% CI, 0.68-1.24) (Marth et al., 2017). The TRINOVA-3 trail assessed the combination of trebananib with paclitaxel and carboplatin in 1015 advanced OC patients. However, no significant improvement was observed in PFS compared with placebo group (15.9 vs. 15.0 months; HR, 0.93; 95%CI, 0.79-1.09; *p* = 0.36). No new safety signals were produced, either Vergote et al. (2019a).

To summarize, the TRINOVA-1 trail showed that trebananib significantly improved PFS in recurrent OC compared with paclitaxel alone. The TRINOVA-2 trail compared paclitaxel plus placebo or paclitaxel plus trebananib in recurrent OC, and the PFS was modestly improved but no significant difference. The TRINOVA-3 trail indicated that trebananib + carboplatin + paclitaxel failed to improve PFS of advanced OC patients compared with placebo group. Based on the available studies and in the absence of effective biomarkers, trebananib possessed an adequate safety profile, but its efficacy in the selected OC population was not significant.

## 5 Biomarkers of anti-angiogenic therapy in OC

**FIGURE 2:**
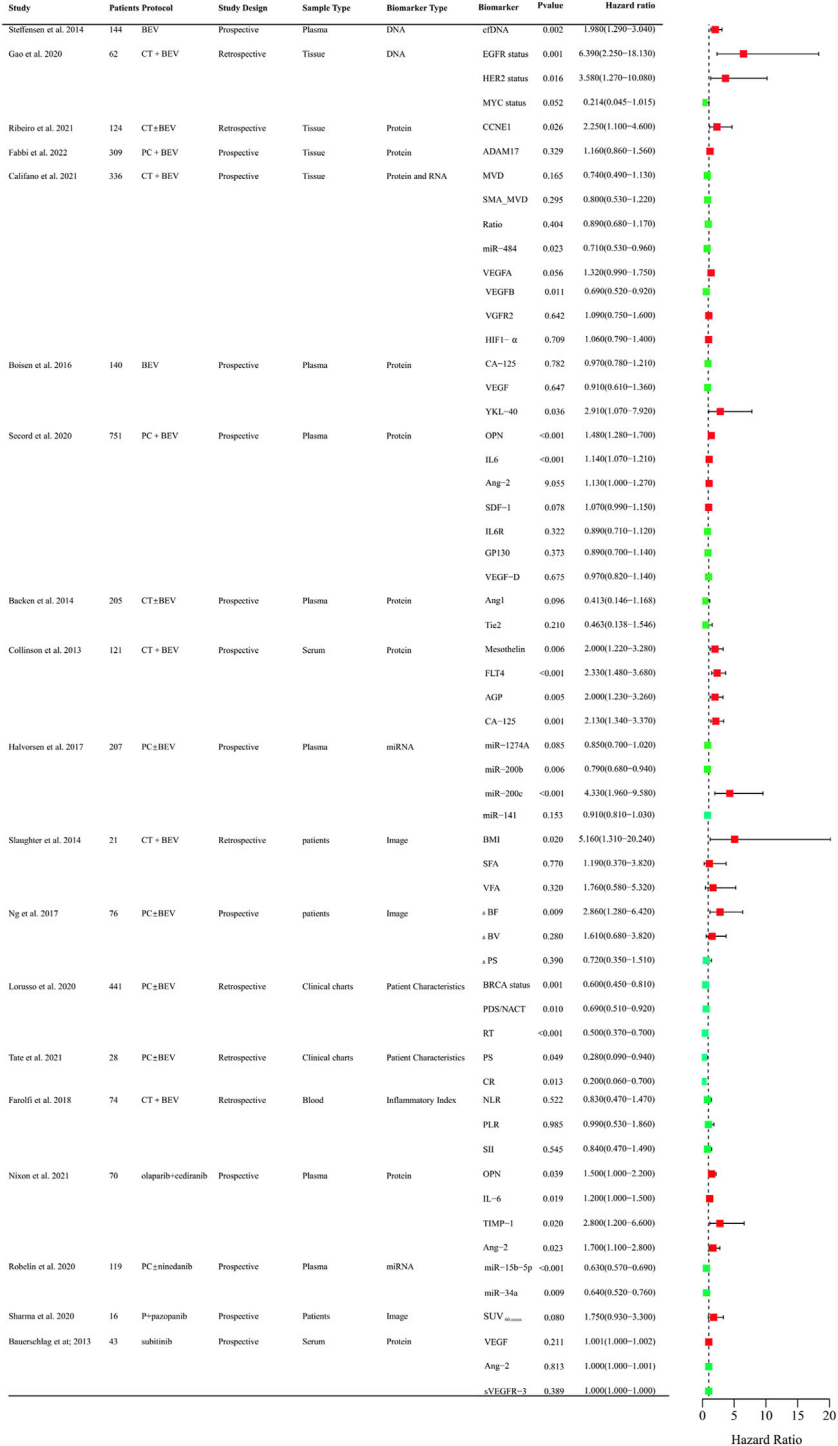
Clinical trials assessing biomarkers in relation to PFS of anti-angiogenic drugs in OC. BEV: Bevacizumab; CT: Chemotherapy; PC:TAXOL and CBP; DNA, Deoxyribonucleic Acid; RNA, Ribonucleic Acid; cfDNA, cell-free DNA; EGFR, Epidermal Growth Factor Receptor; HER2, Human Epidermal GrowthFactor Receptor 2; MYC, MYC Proto-Oncogene; CCNE1, Cyclin E1; ADAM17, a disintegrin and metalloprotease 17; MVD, microvessel density; SMA_MVD: Alfa-Smooth Muscle Actin + microvessel density; Ratio, α-SMA + MVD/MVD ratio; miRNA, microRNA; VEGFA, vascular endothelial growth factor A; VEGFB, vascular endothelial growth factor B; HIF-a, Hypoxia-Inducible Factor 1-alpha; OPN, osteopontin; SDF-1, stromal cell–derived factor-1; IL6R, IL6 receptor; FLT4, fms-like tyrosine kinase-4; AGP, a 1 -acid glycoprotein; BMI, body mass index; VFA, visceral fat area; SFA, subcutaneous fat area; ΔBF, change of Tumor Blood Flow; ΔBV, change of Tumor Blood Volume; ΔPS, change of Vessel Permeability Surface Product; PDS/NACT, Primary Debulking Surgery/Neoadjuvant chemotherapy; RT, Residual Tumor; PS, Performance Status; CR, Completeness of resection; NLR, neutrophil-to-lymphocyte ratio; PLR, platelet-to-lymphocyte ratio; SII, systemic immune inflammation index.

**FIGURE 3:**
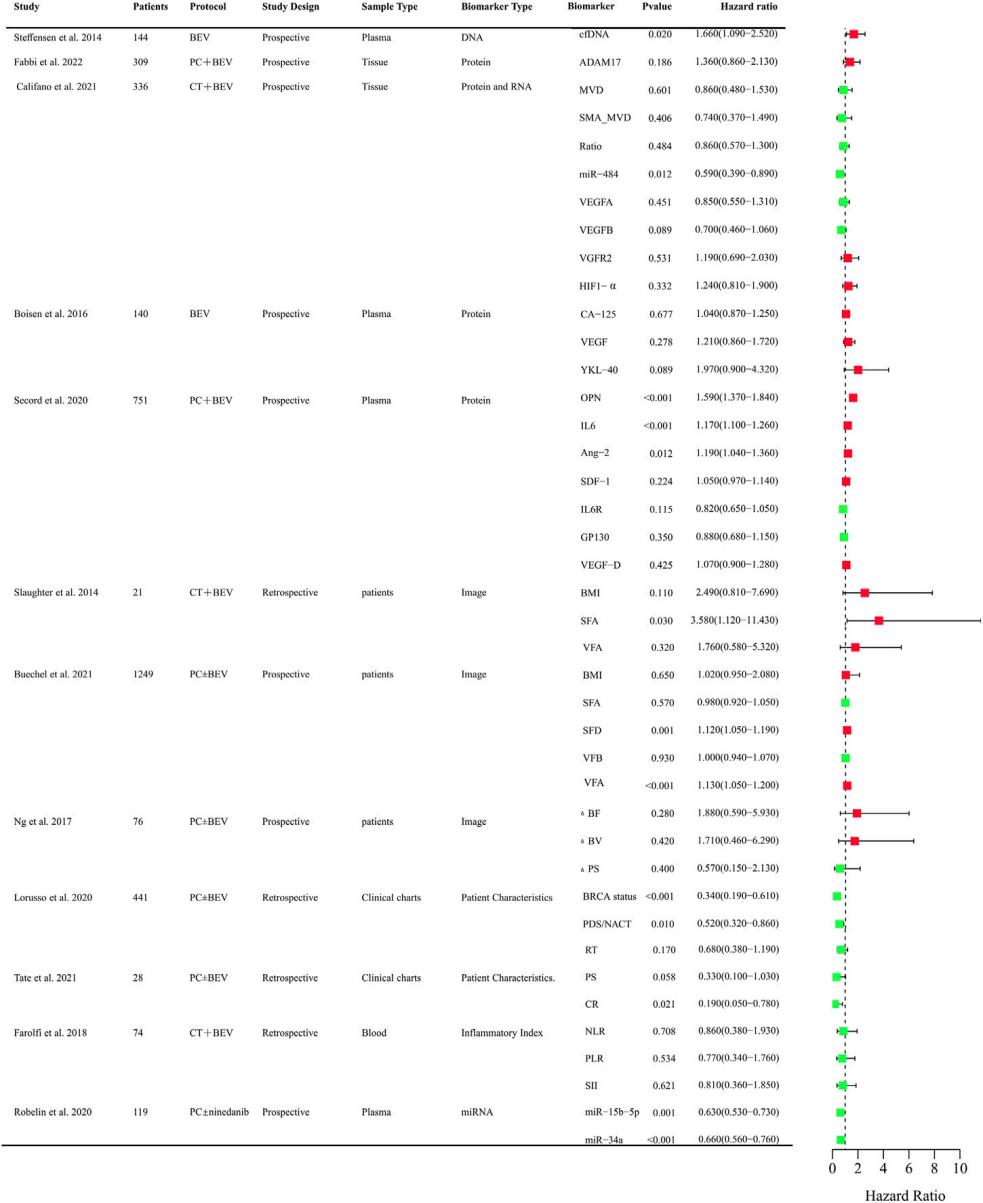
Clinical trials assessing biomarkers in relation to OS of anti-angiogenic drugs in OC. BEV: Bevacizumab; CT: Chemotherapy; PC:TAXOL and CBP; DNA, Deoxyribonucleic Acid; RNA, Ribonucleic Acid; cfDNA, cell-free DNA; ADAM17, a disintegrin and metalloprotease 17; MVD, microvessel density; SMA_MVD: Alfa-Smooth Muscle Actin + microvessel density; Ratio, α-SMA + MVD/MVD ratio; miRNA, microRNA; VEGFA, vascular endothelial growth factor A; VEGFB, vascular endothelial growth factor B; HIF-a, Hypoxia-Inducible Factor 1-alpha; OPN, osteopontin; SDF-1, stromal cell–derived factor-1; IL6R, IL6 receptor; FLT4, fms-like tyrosine kinase-4; AGP, a 1 -acid glycoprotein; BMI, body mass index; VFA, visceral fat area; SFA, subcutaneous fat area; ΔBF, change of Tumor Blood Flow; ΔBV, change of Tumor Blood Volume; ΔPS, change of Vessel Permeability Surface Product; PDS/NACT, Primary Debulking Surgery/Neoadjuvant chemotherapy; RT, Residual Tumor; PS, Performance Status; CR, Completeness of resection; NLR, neutrophil-to-lymphocyte ratio; PLR, platelet-to-lymphocyte ratio; SII, systemic immune inflammation index; SFD, subcutaneous fat density; VFD, visceral fat density.

Anti-angiogenic agents have demonstrated significant efficacy benefits in OC as single-agent or combination therapy. However, not all patients can benefit from these agents. It is crucial to identify clinical biomarkers to select sensitive population and monitor curative effect of anti-angiogenic drugs. So far, numerous studies focused on the research in OC and provided evidence indicating several predictive values for clinical, radiological, molecular, and gene profiling markers (Supplementary Table S1). The biomarkers related to PFS and OS that assessed in clinical trials were systematically summarized in Figures 2, 3, respectively.

Circulating cell-free DNA was shown to be an independent prognostic importance in multi-resistant epithelial OC patients treated with bevacizumab (Steffensen et al., 2014). Epidermal growth factor receptor (EGFR), BRCA, and human epidermal growth factor receptor-2 (HER2) mutational status might be predictors for PFS of chemotherapy and bevacizumab combination therapy in retrospective studies (Lorusso et al., 2020; Gao et al., 2021). The protein expression of angiogenesis-related genes such as CCNE1, a disintegrin and metalloprotease 17 (ADAM17), mevalonate diphosphate decarboxylase (MVD), SMA_MVD, VEGFA, VEGFB, VGFR2, HIF-1α in tumor tissues was explored (Fabbi et al., 2022). Only CCNE1 and VEGFB were proved to be predictive markers for the efficacy of bevacizumab (Califano et al., 2021; Ribeiro et al., 2021). Circulating plasma or serum proteomic biomarkers are also assessed to their predictive value for PFS and OS. Chitinase three like 1 (YKL-40), osteopontin (OPN), IL-6, Ang-2, Mesothelin (MSLN), fms-like tyrosine kinase-4 (FLT4), Alpha-1 acid glycoprotein (AGP), and cancer antigen 125 (CA-125) might be predictive of therapeutic benefit from bevacizumab (Collinson et al., 2013; Backen et al., 2014; Boisen et al., 2016; Alvarez Secord et al., 2020). OPN, IL-6, TIMP-1, Ang-2 were also correlated with PFS in OC patients treated with olaparib + cediranib (Nixon et al., 2021). VEGR, Ang-2, VEGFR-3 were explored for the predictive value for PFS in Pt resistant or refractory OC patients with the treatment of sunitinib. However, there was no significance (Bauerschlag et al., 2013). Circulating microRNAs were also investigated to identify candidate predictive biomarkers for anti-angiogenic drugs in OC. The level of miR-200b, and miR-200c might be predictive of the effect of treatment with bevacizumab (Halvorsen et al., 2017). Low expression of miR-34a-5p and miR-93-5p were correlated with PFS and OS improvements in OC patients with the treatment of chemotherarpy ± nintedanib (Robelin et al., 2020). As obesity was associated with the level of VEGF, the main target of bevacizumab, adiposity was assessed. The measurements of adiposity such as subcutaneous fat area or density and visceral fat density are likely to be useful biomarkers for PFS or OS (Halvorsen et al., 2017; Buechel et al., 2021). CT perfusion biomarkers such as blood flow may offer early prognostic evidence for patients with newly diagnosed OC and received chemotherapy ± bevacizumab therapy (Ng et al., 2017). Baseline SUV_60,mean_ (mean standardized uptake value at 60 min) was negatively correlated with PFS of patlinum-resistant/refractory OC patients received pazopanib and TAXOL combination therapy, which indicated [^18^F] Fluciclatide-PET uptake parameters may be a predictor of clinical outcome in patients treated with pazopanib (Sharma et al., 2020). Inflammatory Indexes were prognostic markers for OC patients treated with chemotherapy, but not with chemotherapy and bevacizumab (Farolfi et al., 2018). These findings need to be validated in further different races and larger sample sizes. It is still urgent to identify predictive biomarkers in treating OC patients with anti-angiogenic agents.

## 6 Models of anti-angiogenic therapy in OC

Angiogenesis is an outcome of complex signaling involving a plethora of cells, their cellular signal transduction, activation, proliferation, differentiation, as well as their intercellular communication. Zhang et al. provided a comprehensive review of systems biology computational models of angiogenesis at the pathway‐, cell‐, tissue‐, and whole body‐levels, which advanced our understanding of signaling in angiogenesis and delivered new translational insights for human diseases (Zhang et al., 2022). An integrated model of VEGF-Ang-2 cooperation that accurately recapitulates molecular events constituting the angiogenic switch was proposed in ovarian cancer (Zhang et al., 2003). Adhikarla et al. established a computational model to simulate tumor-specific oxygenation changes based on the molecular image data, which incorporating therapeutic effects might serve as a powerful tool for analyzing tumor response to anti-angiogenic therapies (Adhikarla and Jeraj, 2016). Models combining biomarkers with other risk factors are also constructed to predict treatment outcomes of anti-angiogenic agents in OC. Previs et al. found that prior number of chemotherapy regimens, treatment-free interval (TFI), Pt sensitivity, and the presence of ascites were significant predictors of 5-year OS in 312 women with recurrent ovarian cancer treated with bevacizumab and chemotherapy. Based on the multivariate analysis, a nomogram for OS was constructed, which could provide insight to those women who will benefit the most while avoiding excessive costs and potentially catastrophic toxicities that would ultimately require discontinuation of therapy (Previs et al., 2014). Wang et al. reported three quantitative adiposity-related image feature-based models (multiple linear, logistic and Cox proportional hazards regressions), which provide a useful and Supplementary Information that could yield higher discriminatory power than BMI in predicting the association between adiposity and clinical outcome of EOC patients (including PFS and OS) treated with maintenance bevacizumab-based chemotherapy (Wang et al., 2016). Sostelly. et al. constructed an OS model combining tumor kinetics metrics describing the change in tumor size over time in Pt-resistant OC (PROC) patients treated with chemotherapy and bevacizumab, which could effectively help to simulate and optimize future trials in PROC population (Sostelly and Mercier, 2019).

## 7 Mechanisms of therapy resistance and adverse reaction

Despite the ever-growing number of anti-angiogenic drugs applied in clinical practice, the survival benefits to date have been quite limited, which only temporarily inhibiting tumor development before drug resistance occurs.

In OC, the vast majority of patients have innate or acquired resistance to anti-angiogenic therapy and eventually recurrence (Ellis and Hicklin, 2008). Even a small proportion of patients could benefit from bevacizumab, the effective duration is relatively short (only 3–8 months with monotherapy). There are several explanations for the modest efficacy, like the adoption of alternative patterns of angiogenesis by the tumor and the development of resistance mechanisms. In case of the high expense, adverse reactions and modest clinical benefit of anti-angiogenic drugs, an insight knowledge of resistance mechanisms and the exploration of reliable predictive biomarkers are in urgent needs to provide a basis for prolonging survival and overcoming resistance (Jin et al., 2022).

Both intrinsic and acquired resistance are considered the major leading to the therapeutic failure of anti-angiogenic agents. The most frequently proposed mechanism is the increase in tumor hypoxia levels caused by anti-angiogenic therapy. Anti-angiogenic agents aggravate intra-tumoral hypoxia and the abnormal upregulation of HIF-1α, this further stimulates the production of angiogenic factors like FGF, ANGPT2, and IL-8, eventually leading to therapy resistance and higher risk of disease progression (Casanovas et al., 2005; Huang et al., 2010; Rigamonti et al., 2014). HIF-1 may be a promising target to improve chemoradiotherapy sensitivity and patient prognosis, upregulation of which greatly enhanced tumor angiogenesis, malignant progression as well as apoptosis resistance. However, there are no clinical studies focused on HIF-1 protein inhibitors yet (Bhattarai et al., 2018). Secondly, when the VEGF/VEGFR pathway is inhibited, other VEGF -independent angiogenic mechanisms such as ANG1, ANGPT-2, FGF-2, IL-8, Dll4/Notch and miRNA46 will be compensatively upregulated, ultimately causing resistance to anti-VEGF drugs (Liu et al., 2021). Thirdly, the heterogeneity of tumor cells is an important endogenous resistance mechanism of anti-angiogenic therapy. Heterogeneity in tumor vasculature itself leads to the differential requirement for VEGF. Among the different types of the blood vessel, the first-formed mother vessels and glomeruloid microvascular proliferations have a high response to anti-VEGF therapy, while the “late” formed capillaries, vascular malformations, feeder arteries, and draining veins are relatively insensitive (Nagy and Dvorak, 2012). Therefore, individual differences, the proportion of vascular subtypes varies in diverse tumor tissues, different ratios of VEGF-dependent and -independent angiogenesis all contribute to resistance to anti-angiogenic agents. Fourth, long-term anti-angiogenic therapy would result in widespread vascular morphological alterations *via* the regulation of pro-angiogenic factors, and the remodeled neovascularization structure results in resistance to existing anti-angiogenic drugs (Huang et al., 2004).

## 8 Combining with immunotherapy

Combination therapy holds great promise in overcoming resistance and enhancing the antitumor efficacy of anti-angiogenic drugs. Immune checkpoint inhibitors (ICIs) exert anticancer effects by reactivating exhausted or dysfunctional T-cells (Mellman et al., 2011; Topalian et al., 2016). Monoclonal antibodies targeting cytotoxic T lymphocyte-associated protein 4 (CTLA-4), programmed cell death protein (PD-1) and its ligand PD-L1 are the most wildly used ICIs. However, ICIs alone showed limited efficacy in advanced or recurrent OC, with an overall response rate (ORR) between 5.9% and 22.2% (Brahmer et al., 2012; Hamanishi et al., 2015; Liu et al., 2019b; Disis et al., 2019; Varga et al., 2019; Nishio et al., 2020; Hamanishi et al., 2021). A phase III study (JAVELIN Ovarian 200) showed that neither monotherapy nor combination of avelumab with chemotherapy improved PFS or OS in patients with platinum-resistant or platinum-refractory OC (Pujade-Lauraine et al., 2021).

The antitumor effect of immunotherapy relies on the accumulation of immune effector cells in tumor microenvironment (TME). The anti-angiogenic therapy-mediated tumor vascular normalization effectively increases the infiltration of immune effector cells in TME and promotes the reprogramming of intrinsically immunosuppressive TME into immune supportive one (Fukumura et al., 2018). Anti-angiogenic therapy also ameliorates antitumor immunity by inhibiting multiple immunosuppressive properties of angiogenesis (Huinen et al., 2021). On the contrary, ICIs-activated immunity improves anti-angiogenic efficiency by reducing the expression of angiogenic factors and alleviating hypoxia conditions (Song et al., 2020).

Mechanism studies have explained the immunosuppressive function of VEGF. For example, VEGF inhibits the maturation and differentiation of dendritic cells through NF-kB signaling pathway (Oyama et al., 1998; Curiel et al., 2003; Huang et al., 2007). It also upregulates the expression of PD-L1, thus inhibiting the antigen presentation function of dendritic cells, and further the activation and expansion of T-cells (Alfaro et al., 2009). Besides, VEGF inhibits the differentiation of monocytes into dendritic cells, which can be reversed by bevacizumab or sorafenib treatment (Motz and Coukos, 2011). VEGF-activated VEGFR-2 stimulates the expression of immune checkpoint molecules including PD-1, T-cell immunoglobulin mucin receptor 3 (TIM-3), and cytotoxic T lymphocyte antigen 4 (CTLA-4) on CD8^+^ cells, resulting in the exhaustion of CD8^+^cytotoxic T-cells (Burger et al., 2011; Perren et al., 2011; Fuh et al., 2015). Moreover, VEGF facilitates the proliferation of Tregs, thereby inhibiting anti-tumor immunity and promoting the occurrence and tumor development (Pennington and Swisher, 2012; Patch et al., 2015). In addition, targeting VEGF/VEGFR can also enhance immunotherapy efficacy by upregulating adhesion molecules and chemokines that are critical for the capture and transendothelial migration of T-cells (Georganaki et al., 2018; Khan and Kerbel, 2018). In view of the demonstrated antitumor efficacy, the FDA has approved the combination of anti-angiogenic agents with ICIs for certain malignancies.

Improved antitumor efficacy and prolonged survival were observed in many clinical trials following the combination of ICIs with anti-angiogenic agents (Song et al., 2020). The combination of bevacizumab and ICIs has been evaluated in phase I and II clinical trials, and the ORR was between 15% and 32%, which was significantly higher than ICIs alone (Langenkamp et al., 2015; Liu et al., 2019c; González-Martín et al., 2020; Moroney et al., 2020). A phase Ib study in platinum-resistant OC showed that the ORR of atezolizumab plus bevacizumab was 15% (Moroney et al., 2020). Another phase II study in relapsed EOC demonstrated that the combination of nivolumab with bevacizumab had an ORR of 40.0% (19.1%-64.0%) and 16.7% (95% CI 3.6%-41.4%) in the platinum-sensitive and -resistant group, respectively (Liu et al., 2019c). In addition, LEAP-005 phase II study evaluated the efficacy and safety of lenvatinib and pembrolizumab (a PD-1 immune checkpoint inhibitor) in patients with OC. The combination reached an ORR of 32% with manageable adverse events (González-Martín et al., 2020)*.*

In conclusion, co-applied ICIs with anti-angiogenic agents has shown satisfactory efficacy in several malignancies. However, several obstacles still exist, like low tumor penetrance and increased adverse reactions. New agents, such as engineered antibodies, may help provide safer and more effective therapies (Anderson et al., 2022).

## 9 Conclusion and prospect

The limitations in the use of anti-angiogenic therapy may be in part related to two main factors. First, the exact mechanisms of angiogenesis and therapeutic resistance remain unclear. Secondly, the abrogation of blood supply also limits the effective transport of antineoplastic agents inside the tumor, thus weaken their anti-tumor effect. The vast majority of clinical studies focused on bevacizumab suggested a meaningful improvement in PFS of recurrent OC patients, regardless of the Pt sensitivity. Similarly, anti-anti-angiogenic drugs targeting TKIs, including sorafenib, pazopanib, cediranib, and nintedanib also exhibited satisfactory improvements in the PFS of OC. However, only a few studies reported significant improvements in the OS of OC patients. In addition, bevacizumab exerted its effectiveness in only a small proportion of patients, while no reliable predictive biomarkers have been identified and validated for more precise treatment with bevacizumab. Regarding the obvious toxicity and high cost, biomarkers are urgent and crucial for selecting patients with a higher possibility to benefit from anti-angiogenic therapy.

## Glossary

ABCB1: ATP binding cassette subfamily B member 1
ADAM17: a disintegrin and metalloprotease 17
AGP: Alpha-1 acid glycoprotein
AKT: protein kinase B
ANGPTs: angiopoietins
APLN: Apelin
APLNR: G protein-coupled receptor APJ
BRCA: breast cancer susceptibility gene
CA-125: cancer antigen 125
CAFs: cancer associated fibroblasts
CBP: carboplatin
CCNE1: cyclin E1
c-MET: hepatocyte growth factor receptor
CTLA-4: cytotoxic T lymphocyte-associated protein 4
EC: endothelial cell
EGFR: epidermal growth factor receptor
EMA: European Medicines Agency
EOC: epithelial ovarian cancer
ERK: extracellular signal-regulated kinase
FDA: Food and Drug Administration
FGFs: fibroblast growth factors
FLT4: fms-like tyrosine kinase-4
HER2: human epidermal growth factor receptor-2
HGF: hepatocyte growth factor
HIF-1α: hypoxia-inducible factor -1α
IGF-1: insulin-like growth factor 1
IL-6: interleukin 6
ICIs: Immune checkpoint inhibitors
IP3: inositol 1,4,5-triphosphate
JAK: Janus kinase
Lck: lymphocyte-specific protein tyrosine kinase
Lyn: tyrosine-protein kinase Lyn
MAPK: mitogen-activated protein kinase
MMP-2: mitochondrial membrane potential-2
MSLN: Mesothelin
MVD: mevalonate diphosphate decarboxylase
NRP1: neuropilin 1; OC, ovarian cancer
OPN: osteopontin
ORR: overall response rate
OS: overall survival
PARP: poly (ADP‐ribose) polymerase
PDGF: platelet-derived growth factor
PD-1: programmed cell death protein
PFS: progression-free survival
PI3K: phosphatidylinositol 3-kinase
PLC: phospsholipase-C
PLC-γ: Phospholipase C-γ
PLD: pegylated liposomal doxorubicin
PlGF: placental growth factor
Pt: platinum
RTK: receptor tyrosine kinases
RTKi: receptor tyrosine kinase inhibitor
Src: proto-oncogene tyrosine-protein kinase Src
STAT: signal transducing activator of transcription
SUV: standardized uptake value
TAMs: tumor-associated macrophages
TAXOL: paclitaxel
TFI: treatment-free interval
TKI: tyrosine kinase inhibitor
TPT: topotecan
uPA: urokinase plasminogen activator
VEGF: vascular endothelial growth factor
YKL-40: Chitinase three like 1

## References

[B1] Abdollahi A. Hahnfeldt P. Maercker C. Grone H. J. Debus J. Ansorge W. (2004). Endostatin's antiangiogenic signaling network. Mol. Cell 13, 649–663. 10.1016/s1097-2765(04)00102-9 15023336

[B2] Adhikarla V. Jeraj R. (2016). An imaging-based computational model for simulating angiogenesis and tumour oxygenation dynamics. Phys. Med. Biol. 61, 3885–3902. 10.1088/0031-9155/61/10/3885 27117345 PMC6284397

[B3] Agarwal R. Kaye S. B. (2003). Ovarian cancer: Strategies for overcoming resistance to chemotherapy. Nat. Rev. Cancer 3, 502–516. 10.1038/nrc1123 12835670

[B4] Aghajanian C. Blank S. V. Goff B. A. Judson P. L. Teneriello M. G. Husain A. (2012). Oceans: A randomized, double-blind, placebo-controlled phase III trial of chemotherapy with or without bevacizumab in patients with platinum-sensitive recurrent epithelial ovarian, primary peritoneal, or fallopian tube cancer. J. Clin. Oncol. 30, 2039–2045. 10.1200/JCO.2012.42.0505 22529265 PMC3646321

[B5] Akai J. Halley P. A. Storey K. G. (2005). FGF-dependent Notch signaling maintains the spinal cord stem zone. Genes Dev. 19, 2877–2887. 10.1101/gad.357705 16287717 PMC1315394

[B6] Alam S. M. Fujimoto J. Jahan I. Sato E. Tamaya T. (2008). Coexpression of EphB4 and ephrinB2 in tumour advancement of ovarian cancers. Br. J. Cancer 98, 845–851. 10.1038/sj.bjc.6604216 18231102 PMC2259170

[B7] Alfaro C. Suarez N. Gonzalez A. Solano S. Erro L. Dubrot J. (2009). Influence of bevacizumab, sunitinib and sorafenib as single agents or in combination on the inhibitory effects of VEGF on human dendritic cell differentiation from monocytes. Br. J. Cancer 100, 1111–1119. 10.1038/sj.bjc.6604965 19277038 PMC2670006

[B8] Alvarez Secord A. Bell Burdett K. Owzar K. Tritchler D. Sibley A. B. Liu Y. (2020). Predictive blood-based biomarkers in patients with epithelial ovarian cancer treated with carboplatin and paclitaxel with or without bevacizumab: Results from GOG-0218. Clin. Cancer Res. 26, 1288–1296. 10.1158/1078-0432.CCR-19-0226 31919136 PMC7073274

[B9] Anderson T. S. Wooster A. L. Piersall S. L. Okpalanwaka I. F. Lowe D. B. (2022). Disrupting cancer angiogenesis and immune checkpoint networks for improved tumor immunity. Semin. Cancer Biol. 86, 981–996. 10.1016/j.semcancer.2022.02.009 35149179 PMC9357867

[B11] Arjaans M. Oude Munnink T. H. Oosting S. F. Terwisscha van Scheltinga A. G. Gietema J. A. Garbacik E. T. (2013). Bevacizumab-induced normalization of blood vessels in tumors hampers antibody uptake. Cancer Res. 73, 3347–3355. 10.1158/0008-5472.CAN-12-3518 23580572

[B12] Backen A. Renehan A. G. Clamp A. R. Berzuini C. Zhou C. Oza A. (2014). The combination of circulating Ang1 and Tie2 levels predicts progression-free survival advantage in bevacizumab-treated patients with ovarian cancer. Clin. Cancer Res. 20, 4549–4558. 10.1158/1078-0432.CCR-13-3248 24947924 PMC4154862

[B13] Bagley R. G. Ren Y. Weber W. Yao M. Kurtzberg L. Pinckney J. (2011). Placental growth factor upregulation is a host response to antiangiogenic therapy. Clin. Cancer Res. 17, 976–988. 10.1158/1078-0432.CCR-10-2687 21343374

[B14] Bamberger E. S. Perrett C. W. (2002). Angiogenesis in epithelian ovarian cancer. Mol. Pathol. 55, 348–359. 10.1136/mp.55.6.348 12456770 PMC1187269

[B15] Banerjee S. Kaye S. (2011). The role of targeted therapy in ovarian cancer. Eur. J. Cancer 47 (3), S116–S130. 10.1016/S0959-8049(11)70155-1 21943965

[B16] Bartoschek M. Pietras K. (2018). PDGF family function and prognostic value in tumor biology. Biochem. Biophys. Res. Commun. 503, 984–990. 10.1016/j.bbrc.2018.06.106 29932922

[B17] Bauerschlag D. O. Hilpert F. Meier W. Rau J. Meinhold-Heerlein I. Maass N. (2013). Evaluation of potentially predictive markers for anti-angiogenic therapy with sunitinib in recurrent ovarian cancer patients. Transl. Oncol. 6, 305–310. 10.1593/tlo.13205 23730410 PMC3660799

[B18] Bauerschlag D. O. Schem C. Tiwari S. Egberts J. H. Weigel M. T. Kalthoff H. (2010). Sunitinib (SU11248) inhibits growth of human ovarian cancer in xenografted mice. Anticancer Res. 30, 3355–3360. 20944108

[B19] Baumann K. H. du Bois A. Meier W. Rau J. Wimberger P. Sehouli J. (2012). A phase II trial (AGO 2.11) in platinum-resistant ovarian cancer: A randomized multicenter trial with sunitinib (SU11248) to evaluate dosage, schedule, tolerability, toxicity and effectiveness of a multitargeted receptor tyrosine kinase inhibitor monotherapy. Ann. Oncol. 23, 2265–2271. 10.1093/annonc/mds003 22377563

[B20] Berta J. Kenessey I. Dobos J. Tovari J. Klepetko W. Jan Ankersmit H. (2010). Apelin expression in human non-small cell lung cancer: Role in angiogenesis and prognosis. J. Thorac. Oncol. 5, 1120–1129. 10.1097/JTO.0b013e3181e2c1ff 20581707

[B21] Bhattarai D. Xu X. Lee K. (2018). Hypoxia-inducible factor-1 (HIF-1) inhibitors from the last decade (2007 to 2016): A "structure-activity relationship" perspective. Med. Res. Rev. 38, 1404–1442. 10.1002/med.21477 29278273

[B22] Biagi J. J. Oza A. M. Chalchal H. I. Grimshaw R. Ellard S. L. Lee U. (2011). A phase II study of sunitinib in patients with recurrent epithelial ovarian and primary peritoneal carcinoma: An NCIC clinical trials group study. Ann. Oncol. 22, 335–340. 10.1093/annonc/mdq357 20705911

[B23] Bodnar L. Gornas M. Szczylik C. (2011). Sorafenib as a third line therapy in patients with epithelial ovarian cancer or primary peritoneal cancer: A phase II study. Oncol 123, 33–36. 10.1016/j.ygyno.2011.06.019 21723597

[B24] Bohndiek S. E. Sasportas L. S. Machtaler S. Jokerst J. V. Hori S. Gambhir S. S. (2015). Photoacoustic tomography detects early vessel regression and normalization during ovarian tumor response to the antiangiogenic therapy trebananib. J. Nucl. Med. 56, 1942–1947. 10.2967/jnumed.115.160002 26315834 PMC5612481

[B25] Boisen M. K. Madsen C. V. Dehlendorff C. Jakobsen A. Johansen J. S. Steffensen K. D. (2016). The prognostic value of plasma YKL-40 in patients with chemotherapy-resistant ovarian cancer treated with bevacizumab. Int. J. Gynecol. Cancer 26, 1390–1398. 10.1097/IGC.0000000000000798 27648712

[B26] Brahmer J. R. Tykodi S. S. Chow L. Q. Hwu W. J. Topalian S. L. Hwu P. (2012). Safety and activity of anti-PD-L1 antibody in patients with advanced cancer. N. Engl. J. Med. 366, 2455–2465. 10.1056/NEJMoa1200694 22658128 PMC3563263

[B27] Brantley D. M. Cheng N. Thompson E. J. Lin Q. Brekken R. A. Thorpe P. E. (2002). Soluble Eph A receptors inhibit tumor angiogenesis and progression *in vivo* . Oncogene 21, 7011–7026. 10.1038/sj.onc.1205679 12370823

[B28] Bray F. Ferlay J. Soerjomataram I. Siegel R. L. Torre L. A. Jemal A. (2018). Global cancer statistics 2018: GLOBOCAN estimates of incidence and mortality worldwide for 36 cancers in 185 countries. CA Cancer J. Clin. 68, 394–424. 10.3322/caac.21492 30207593

[B29] Brunckhorst M. K. Xu Y. Lu R. Yu Q. (2014). Angiopoietins promote ovarian cancer progression by establishing a procancer microenvironment. Am. J. Pathol. 184, 2285–2296. 10.1016/j.ajpath.2014.05.006 25043619 PMC4116697

[B30] Buechel M. E. Enserro D. Burger R. A. Brady M. F. Wade K. Secord A. A. (2021). Correlation of imaging and plasma based biomarkers to predict response to bevacizumab in epithelial ovarian cancer (EOC). Gynecol. Oncol. 161, 382–388. 10.1016/j.ygyno.2021.02.032 33712274 PMC8327185

[B31] Burbridge M. F. Bossard C. J. Saunier C. Fejes I. Bruno A. Leonce S. (2013). S49076 is a novel kinase inhibitor of MET, AXL, and FGFR with strong preclinical activity alone and in association with bevacizumab. Mol. Cancer Ther. 12, 1749–1762. 10.1158/1535-7163.MCT-13-0075 23804704

[B32] Burger R. A. Brady M. F. Bookman M. A. Fleming G. F. Monk B. J. Huang H. (2011). Incorporation of bevacizumab in the primary treatment of ovarian cancer. N. Engl. J. Med. 365, 2473–2483. 10.1056/nejmoa1104390 22204724

[B33] Burger R. A. (2011). Overview of anti-angiogenic agents in development for ovarian cancer. Gynecol. Oncol. 121, 230–238. 10.1016/j.ygyno.2010.11.035 21215996

[B34] Burger R. A. Sill M. W. Monk B. J. Greer B. E. Sorosky J. I. (2007). Phase II trial of bevacizumab in persistent or recurrent epithelial ovarian cancer or primary peritoneal cancer: A gynecologic oncology group study. J. Clin. Oncol. 25, 5165–5171. 10.1200/JCO.2007.11.5345 18024863

[B35] Byron S. A. Gartside M. G. Wellens C. L. Goodfellow P. J. Birrer M. J. Campbell I. G. (2010). FGFR2 mutations are rare across histologic subtypes of ovarian cancer. Gynecol. Oncol. 117, 125–129. 10.1016/j.ygyno.2009.12.002 20106510

[B36] Califano D. Gallo D. Rampioni Vinciguerra G. L. De Cecio R. Arenare L. Signoriello S. (2021). Evaluation of angiogenesis-related genes as prognostic biomarkers of bevacizumab treated ovarian cancer patients: Results from the phase IV mito16a/ManGO OV-2 translational study. Cancers (Basel) 13, 5152. 10.3390/cancers13205152 34680301 PMC8533892

[B37] Campbell N. E. Greenaway J. Henkin J. Moorehead R. A. Petrik J. (2010). The thrombospondin-1 mimetic ABT-510 increases the uptake and effectiveness of cisplatin and paclitaxel in a mouse model of epithelial ovarian cancer. Neoplasia 12, 275–283. 10.1593/neo.91880 20234821 PMC2838444

[B38] Campos S. M. Penson R. T. Matulonis U. Horowitz N. S. Whalen C. Pereira L. (2013). A phase II trial of Sunitinib malate in recurrent and refractory ovarian, fallopian tube and peritoneal carcinoma. Gynecol. Oncol. 128, 215–220. 10.1016/j.ygyno.2012.07.126 22885865

[B39] Cannistra S. A. Matulonis U. A. Penson R. T. Hambleton J. Dupont J. Mackey H. (2007). Phase II study of bevacizumab in patients with platinum-resistant ovarian cancer or peritoneal serous cancer. J. Clin. Oncol. 25, 5180–5186. 10.1200/JCO.2007.12.0782 18024865

[B40] Cantanhede I. G. de Oliveira J. R. M. (2017). PDGF family expression in glioblastoma multiforme: Data compilation from ivy glioblastoma atlas project database. Sci. Rep. 7, 15271. 10.1038/s41598-017-15045-w 29127351 PMC5681588

[B41] Cao Y. (2013). Multifarious functions of PDGFs and PDGFRs in tumor growth and metastasis. Trends Mol. Med. 19, 460–473. 10.1016/j.molmed.2013.05.002 23773831

[B42] Carmeliet P. Jain R. K. (2011). Molecular mechanisms and clinical applications of angiogenesis. Nature 473, 298–307. 10.1038/nature10144 21593862 PMC4049445

[B43] Carmeliet P. Jain R. K. (2011). Principles and mechanisms of vessel normalization for cancer and other angiogenic diseases. Nat. Rev. Drug Discov. 10, 417–427. 10.1038/nrd3455 21629292

[B44] Casanovas O. Hicklin D. J. Bergers G. Hanahan D. (2005). Drug resistance by evasion of antiangiogenic targeting of VEGF signaling in late-stage pancreatic islet tumors. Cancer Cell 8, 299–309. 10.1016/j.ccr.2005.09.005 16226705

[B45] Chae S. S. Kamoun W. S. Farrar C. T. Kirkpatrick N. D. Niemeyer E. de Graaf A. M. (2010). Angiopoietin-2 interferes with anti-VEGFR2-induced vessel normalization and survival benefit in mice bearing gliomas. Clin. Cancer Res. 16, 3618–3627. 10.1158/1078-0432.CCR-09-3073 20501615 PMC2905497

[B46] Chan J. K. Brady W. Monk B. J. Brown J. Shahin M. S. Rose P. G. (2018). A phase II evaluation of sunitinib in the treatment of persistent or recurrent clear cell ovarian carcinoma: An NRG Oncology/Gynecologic Oncology Group Study (GOG-254). Gynecol. Oncol. 150, 247–252. 10.1016/j.ygyno.2018.05.029 29921512 PMC6235144

[B47] Chekerov R. Hilpert F. Mahner S. El-Balat A. Harter P. De Gregorio N. (2018). Sorafenib plus topotecan versus placebo plus topotecan for platinum-resistant ovarian cancer (TRIAS): A multicentre, randomised, double-blind, placebo-controlled, phase 2 trial. Lancet Oncol. 19, 1247–1258. 10.1016/s1470-2045(18)30372-3 30100379

[B48] Cheng N. Brantley D. Fang W. B. Liu H. Fanslow W. Cerretti D. P. (2003). Inhibition of VEGF-dependent multistage carcinogenesis by soluble EphA receptors. Neoplasia 5, 445–456. 10.1016/s1476-5586(03)80047-7 14670182 PMC1502614

[B49] Chiron M. Bagley R. G. Pollard J. Mankoo P. K. Henry C. Vincent L. (2014). Differential antitumor activity of aflibercept and bevacizumab in patient-derived xenograft models of colorectal cancer. Mol. Cancer Ther. 13, 1636–1644. 10.1158/1535-7163.MCT-13-0753 24688047

[B50] Cloughesy T. Finocchiaro G. Belda-Iniesta C. Recht L. Brandes A. A. Pineda E. (2017). Randomized, double-blind, placebo-controlled, multicenter phase II study of onartuzumab plus bevacizumab versus placebo plus bevacizumab in patients with recurrent glioblastoma: Efficacy, safety, and hepatocyte growth factor and O(6)-methylguanine-DNA methyltransferase biomarker analyses. J. Clin. Oncol. 35, 343–351. 10.1200/JCO.2015.64.7685 27918718

[B51] Coleman R. L. Brady M. F. Herzog T. J. Sabbatini P. Armstrong D. K. Walker J. L. (2017). Bevacizumab and paclitaxel-carboplatin chemotherapy and secondary cytoreduction in recurrent, platinum-sensitive ovarian cancer (NRG oncology/gynecologic oncology group study GOG-0213): A multicentre, open-label, randomised, phase 3 trial. Lancet Oncol. 18, 779–791. 10.1016/S1470-2045(17)30279-6 28438473 PMC5715461

[B52] Collinson F. Hutchinson M. Craven R. A. Cairns D. A. Zougman A. Wind T. C. (2013). Predicting response to bevacizumab in ovarian cancer: A panel of potential biomarkers informing treatment selection. Clin. Cancer Res. 19, 5227–5239. 10.1158/1078-0432.CCR-13-0489 23935036 PMC3780518

[B53] Colombo N. Tomao F. Benedetti Panici P. Nicoletto M. O. Tognon G. Bologna A. (2022). Randomized phase II trial of weekly paclitaxel vs. cediranib-olaparib (continuous or intermittent schedule) in platinum-resistant high-grade epithelial ovarian cancer. Gynecol. Oncol. 164, 505–513. 10.1016/j.ygyno.2022.01.015 35063281

[B54] Cortez A. J. Tudrej P. Kujawa K. A. Lisowska K. M. (2018). Advances in ovarian cancer therapy. Cancer Chemother. Pharmacol. 81, 17–38. 10.1007/s00280-017-3501-8 29249039 PMC5754410

[B55] Curiel T. J. Wei S. Dong H. Alvarez X. Cheng P. Mottram P. (2003). Blockade of B7-H1 improves myeloid dendritic cell-mediated antitumor immunity. Nat. Med. 9, 562–567. 10.1038/nm863 12704383

[B56] Davidson B. A. Secord A. A. (2014). Profile of pazopanib and its potential in the treatment of epithelial ovarian cancer. Int. J. Womens Health 6, 289–300. 10.2147/IJWH.S49781 24648773 PMC3958497

[B57] De Falco S. (2012). The discovery of placenta growth factor and its biological activity. Exp. Mol. Med. 44, 1–9. 10.3858/emm.2012.44.1.025 22228176 PMC3277892

[B58] Delgado V. M. Nugnes L. G. Colombo L. L. Troncoso M. F. Fernandez M. M. Malchiodi E. L. (2011). Modulation of endothelial cell migration and angiogenesis: A novel function for the "tandem-repeat" lectin galectin-8. FASEB J. 25, 242–254. 10.1096/fj.09-144907 20876211

[B59] Dewangan J. Srivastava S. Mishra S. Divakar A. Kumar S. Rath S. K. (2019). Salinomycin inhibits breast cancer progression via targeting HIF-1α/VEGF mediated tumor angiogenesis *in vitro* and *in vivo* . Biochem. Pharmacol. 164, 326–335. 10.1016/j.bcp.2019.04.026 31028743

[B60] Dewhirst M. W. Ashcraft K. A. (2016). Implications of increase in vascular permeability in tumors by VEGF: A commentary on the pioneering work of harold Dvorak. Cancer Res. 76, 3118–3120. 10.1158/0008-5472.CAN-16-1292 27251086

[B61] Dewhirst M. W. Ong E. T. Braun R. D. Smith B. Klitzman B. Evans S. M. (1999). Quantification of longitudinal tissue pO2 gradients in window chamber tumours: Impact on tumour hypoxia. Br. J. Cancer 79, 1717–1722. 10.1038/sj.bjc.6690273 10206282 PMC2362789

[B62] Dewhirst M. W. Secomb T. W. (2017). Transport of drugs from blood vessels to tumour tissue. Nat. Rev. Cancer 17, 738–750. 10.1038/nrc.2017.93 29123246 PMC6371795

[B63] Dhanabal M. Jeffers M. LaRochelle W. J. Lichenstein H. S. (2005). Angioarrestin: A unique angiopoietin-related protein with anti-angiogenic properties. Biochem. Biophys. Res. Commun. 333, 308–315. 10.1016/j.bbrc.2005.05.134 15950186

[B64] Disis M. L. Taylor M. H. Kelly K. Beck J. T. Gordon M. Moore K. M. (2019). Efficacy and safety of avelumab for patients with recurrent or refractory ovarian cancer: Phase 1b results from the JAVELIN solid tumor trial. JAMA Oncol. 5, 393–401. 10.1001/jamaoncol.2018.6258 30676622 PMC6439837

[B65] Dobrzanski P. Hunter K. Jones-Bolin S. Chang H. Robinson C. Pritchard S. (2004). Antiangiogenic and antitumor efficacy of EphA2 receptor antagonist. Cancer Res. 64, 910–919. 10.1158/0008-5472.can-3430-2 14871820

[B66] Dogra S. Neelakantan D. Patel M. M. Griesel B. Olson A. Woo S. (2021). Adipokine apelin/APJ pathway promotes peritoneal dissemination of ovarian cancer cells by regulating lipid metabolism. Mol. Cancer Res. 19, 1534–1545. 10.1158/1541-7786.MCR-20-0991 34172534 PMC11486291

[B67] du Bois A. Floquet A. Kim J. W. Rau J. del Campo J. M. Friedlander M. (2014). Incorporation of pazopanib in maintenance therapy of ovarian cancer. J. Clin. Oncol. 32, 3374–3382. 10.1200/JCO.2014.55.7348 25225436

[B68] du Bois A. Kristensen G. Ray-Coquard I. Reuss A. Pignata S. Colombo N. (2016). Standard first-line chemotherapy with or without nintedanib for advanced ovarian cancer (AGO-OVAR 12): A randomised, double-blind, placebo-controlled phase 3 trial. Lancet Oncol. 17, 78–89. 10.1016/s1470-2045(15)00366-6 26590673

[B69] du Bois A. Vergote I. Wimberger P. Ray-Coquard I. Harter P. Curtis L. B. (2012). Open-label feasibility study of pazopanib, carboplatin, and paclitaxel in women with newly diagnosed, untreated, gynaecologic tumours: A phase I/II trial of the AGO study group. Br. J. Cancer 106, 629–632. 10.1038/bjc.2011.608 22240783 PMC3322958

[B70] Ellis L. M. Hicklin D. J. (2008). Pathways mediating resistance to vascular endothelial growth factor-targeted therapy. Clin. Cancer Res. 14, 6371–6375. 10.1158/1078-0432.CCR-07-5287 18927275

[B71] Elyashiv O. Ledermann J. Parmar G. Farrelly L. Counsell N. Feeney A. (2021). ICON 9-an international phase III randomized study to evaluate the efficacy of maintenance therapy with olaparib and cediranib or olaparib alone in patients with relapsed platinum-sensitive ovarian cancer following a response to platinum-based chemotherapy. Int. J. Gynecol. Cancer 31, 134–138. 10.1136/ijgc-2020-002073 33097567

[B72] Erber R. Thurnher A. Katsen A. D. Groth G. Kerger H. Hammes H. P. (2004). Combined inhibition of VEGF and PDGF signaling enforces tumor vessel regression by interfering with pericyte-mediated endothelial cell survival mechanisms. FASEB J. 18, 338–340. 10.1096/fj.03-0271fje 14657001

[B73] Fabbi M. Costa D. Russo D. Arenare L. Gaggero G. Signoriello S. (2022). Analysis of A Disintegrin and metalloprotease 17 (ADAM17) expression as a prognostic marker in ovarian cancer patients undergoing first-line treatment plus bevacizumab. Diagn. (Basel) 12, 2118. 10.3390/diagnostics12092118 PMC9498026 36140519

[B74] Farolfi A. Petrone M. Scarpi E. Galla V. Greco F. Casanova C. (2018). Inflammatory indexes as prognostic and predictive factors in ovarian cancer treated with chemotherapy alone or together with bevacizumab. A multicenter, retrospective analysis by the MITO group (MITO 24). Oncol 13, 469–479. 10.1007/s11523-018-0574-1 29948780

[B75] Feng M. Yao G. Yu H. Qing Y. Wang K. (2016). Tumor apelin, not serum apelin, is associated with the clinical features and prognosis of gastric cancer. BMC Cancer 16, 794. 10.1186/s12885-016-2815-y 27733135 PMC5062883

[B76] Franco M. Roswall P. Cortez E. Hanahan D. Pietras K. (2011). Pericytes promote endothelial cell survival through induction of autocrine VEGF-A signaling and Bcl-w expression. Blood 118, 2906–2917. 10.1182/blood-2011-01-331694 21778339 PMC3172806

[B77] Fuh K. C. Secord A. A. Bevis K. S. Huh W. ElNaggar A. Blansit K. (2015). Comparison of bevacizumab alone or with chemotherapy in recurrent ovarian cancer patients. Gynecol. Oncol. 139, 413–418. 10.1016/j.ygyno.2015.06.041 26144600

[B78] Fukumura D. Kloepper J. Amoozgar Z. Duda D. G. Jain R. K. (2018). Enhancing cancer immunotherapy using antiangiogenics: Opportunities and challenges. Nat. Rev. Clin. Oncol. 15, 325–340. 10.1038/nrclinonc.2018.29 29508855 PMC5921900

[B79] Gao J. Li F. Liu Z. Huang M. Chen H. Liao G. (2021). Multiple genetic variants predict the progression-free survival of bevacizumab plus chemotherapy in advanced ovarian cancer: A retrospective study. Med. Baltim. 100, e27130. 10.1097/MD.0000000000027130 PMC8415939 34477158

[B80] Garrido-Laguna I. Krop I. Burris H. A. 3rd Hamilton E. Braiteh F. Weise A. M. (2019). First-in-human, phase I study of PF-06647263, an anti-EFNA4 calicheamicin antibody-drug conjugate, in patients with advanced solid tumors. Int. J. Cancer 145, 1798–1808. 10.1002/ijc.32154 30680712 PMC6875752

[B81] Gavalas N. G. Liontos M. Trachana S. P. Bagratuni T. Arapinis C. Liacos C. (2013). Angiogenesis-related pathways in the pathogenesis of ovarian cancer. Int. J. Mol. Sci. 14, 15885–15909. 10.3390/ijms140815885 23903048 PMC3759892

[B82] Georganaki M. van Hooren L. Dimberg A. (2018). Vascular targeting to increase the efficiency of immune checkpoint blockade in cancer. Front. Immunol. 9, 3081. 10.3389/fimmu.2018.03081 30627131 PMC6309238

[B83] Gerald D. Chintharlapalli S. Augustin H. G. Benjamin L. E. (2013). Angiopoietin-2: An attractive target for improved antiangiogenic tumor therapy. Cancer Res. 73, 1649–1657. 10.1158/0008-5472.CAN-12-4697 23467610

[B84] Ghedini G. C. Ronca R. Presta M. Giacomini A. (2018). Future applications of FGF/FGFR inhibitors in cancer. Expert Rev. Anticancer Ther. 18, 861–872. 10.1080/14737140.2018.1491795 29936878

[B85] González-Martín A. Chung H. Saada-Bouzid E. Yanez E. Senellart H. Cassier P. (2020) Efficacy Saf. lenvatinib plus pembrolizumab patients previously Treat. ovarian cancer multicohort phase 2 LEAP-005 study 30, 2. A1–A2. 10.1016/j.ygyno.2024.04.011 38718741

[B86] Greenberg J. I. Shields D. J. Barillas S. G. Acevedo L. M. Murphy E. Huang J. (2008). A role for VEGF as a negative regulator of pericyte function and vessel maturation. Nature 456, 809–813. 10.1038/nature07424 18997771 PMC2605188

[B87] Grossfeld G. D. Ginsberg D. A. Stein J. P. Bochner B. H. Esrig D. Groshen S. (1997). Thrombospondin-1 expression in bladder cancer: Association with p53 alterations, tumor angiogenesis, and tumor progression. J. Natl. Cancer Inst. 89, 219–227. 10.1093/jnci/89.3.219 9017002

[B88] Hainsworth J. D. Thompson D. S. Bismayer J. A. Gian V. G. Merritt W. M. Whorf R. C. (2015). Paclitaxel/carboplatin with or without sorafenib in the first-line treatment of patients with stage III/IV epithelial ovarian cancer: A randomized phase II study of the sarah cannon research Institute. Cancer Med. 4, 673–681. 10.1002/cam4.376 25556916 PMC4430260

[B89] Hall M. R. Dehbi H. M. Banerjee S. Lord R. Clamp A. Ledermann J. A. (2020). A phase II randomised, placebo-controlled trial of low dose (metronomic) cyclophosphamide and nintedanib (BIBF1120) in advanced ovarian, fallopian tube or primary peritoneal cancer. Gynecol. Oncol. 159, 692–698. 10.1016/j.ygyno.2020.09.048 33077258

[B10] Halvorsen A. R. Kristensen G. Embleton A. Adusei C. Barretina-Ginesta M. P. Beale P. (2017). Evaluation of prognostic and predictive significance of circulating MicroRNAs in ovarian cancer patients. Dis. Markers 2017, 3098542, 10.1155/2017/3098542 28293063 PMC5331307

[B90] Hamanishi J. Mandai M. Ikeda T. Minami M. Kawaguchi A. Murayama T. (2015). Safety and antitumor activity of anti-PD-1 antibody, nivolumab, in patients with platinum-resistant ovarian cancer. J. Clin. Oncol. 33, 4015–4022. 10.1200/JCO.2015.62.3397 26351349

[B91] Hamanishi J. Takeshima N. Katsumata N. Ushijima K. Kimura T. Takeuchi S. (2021). Nivolumab versus gemcitabine or pegylated liposomal doxorubicin for patients with platinum-resistant ovarian cancer: Open-label, randomized trial in Japan (NINJA). J. Clin. Oncol. 39, 3671–3681. 10.1200/JCO.21.00334 34473544 PMC8601279

[B92] Han L. Dong Z. Qiao Y. Kristensen G. B. Holm R. Nesland J. M. (2005). The clinical significance of EphA2 and Ephrin A-1 in epithelial ovarian carcinomas. Gynecol. Oncol. 99, 278–286. 10.1016/j.ygyno.2005.06.036 16061279

[B93] Haunschild C. E. Tewari K. S. (2020). Bevacizumab use in the frontline, maintenance and recurrent settings for ovarian cancer. Future Oncol. 16, 225–246. 10.2217/fon-2019-0042 31746224 PMC7036749

[B94] Heldin C. H. (2013). Targeting the PDGF signaling pathway in tumor treatment. Cell Commun. Signal 11, 97. 10.1186/1478-811X-11-97 24359404 PMC3878225

[B95] Heldin C. H. Westermark B. (1999). Mechanism of action and *in vivo* role of platelet-derived growth factor. Physiol. Rev. 79, 1283–1316. 10.1152/physrev.1999.79.4.1283 10508235

[B96] Heo K. Kim Y. H. Sung H. J. Li H. Y. Yoo C. W. Kim J. Y. (2012). Hypoxia-induced up-regulation of apelin is associated with a poor prognosis in oral squamous cell carcinoma patients. Oral Oncol. 48, 500–506. 10.1016/j.oraloncology.2011.12.015 22285858

[B97] Herath N. I. Spanevello M. D. Sabesan S. Newton T. Cummings M. Duffy S. (2006). Over-expression of Eph and ephrin genes in advanced ovarian cancer: Ephrin gene expression correlates with shortened survival. BMC Cancer 6, 144. 10.1186/1471-2407-6-144 16737551 PMC1501040

[B98] Herzog T. J. Scambia G. Kim B. G. Lhomme C. Markowska J. Ray-Coquard I. (2013). A randomized phase II trial of maintenance therapy with Sorafenib in front-line ovarian carcinoma. Gynecol. Oncol. 130, 25–30. 10.1016/j.ygyno.2013.04.011 23591401

[B99] Hirte H. Lheureux S. Fleming G. F. Sugimoto A. Morgan R. Biagi J. (2015). A phase 2 study of cediranib in recurrent or persistent ovarian, peritoneal or fallopian tube cancer: A trial of the princess margaret, chicago and California phase II consortia. Gynecol. Oncol. 138, 55–61. 10.1016/j.ygyno.2015.04.009 25895616

[B100] Hoffmann M. Fiedor E. Ptak A. (2017). Bisphenol A and its derivatives tetrabromobisphenol A and tetrachlorobisphenol A induce apelin expression and secretion in ovarian cancer cells through a peroxisome proliferator-activated receptor gamma-dependent mechanism. Toxicol. Lett. 269, 15–22. 10.1016/j.toxlet.2017.01.006 28111160

[B101] Huang D. Ding Y. Zhou M. Rini B. I. Petillo D. Qian C. N. (2010). Interleukin-8 mediates resistance to antiangiogenic agent sunitinib in renal cell carcinoma. Cancer Res. 70, 1063–1071. 10.1158/0008-5472.CAN-09-3965 20103651 PMC3719378

[B102] Huang J. Soffer S. Z. Kim E. S. McCrudden K. W. Huang J. New T. (2004). Vascular remodeling marks tumors that recur during chronic suppression of angiogenesis. Mol. Cancer Res. 2, 36–42. 10.1158/1541-7786.36.2.1 14757844

[B103] Huang Y. Chen X. Dikov M. M. Novitskiy S. V. Mosse C. A. Yang L. (2007). Distinct roles of VEGFR-1 and VEGFR-2 in the aberrant hematopoiesis associated with elevated levels of VEGF. Blood 110, 624–631. 10.1182/blood-2007-01-065714 17376891 PMC1924481

[B104] Huinen Z. R. Huijbers E. J. M. van Beijnum J. R. Nowak-Sliwinska P. Griffioen A. W. (2021). Anti-angiogenic agents - overcoming tumour endothelial cell anergy and improving immunotherapy outcomes. Nat. Rev. Clin. Oncol. 18, 527–540. 10.1038/s41571-021-00496-y 33833434

[B105] Jeanne A. Sarazin T. Charle M. Moali C. Fichel C. Boulagnon-Rombi C. (2021). Targeting ovarian carcinoma with TSP-1:CD47 antagonist TAX2 activates anti-tumor immunity. Cancers (Basel) 13, 5019. 10.3390/cancers13195019 34638503 PMC8508526

[B106] Jiang B. H. Liu L. Z. (2009). PI3K/PTEN signaling in angiogenesis and tumorigenesis. Adv. Cancer Res. 102, 19–65. 10.1016/S0065-230X(09)02002-8 19595306 PMC2933405

[B107] Jin C. Yuan M. Bu H. Jin C. (2022). Antiangiogenic strategies in epithelial ovarian cancer: Mechanism, resistance, and combination therapy. J. Oncol. 2022, 4880355. 10.1155/2022/4880355 35466318 PMC9019437

[B108] Jing Y. Lu H. Wu K. Subramanian I. V. Ramakrishnan S. (2011). Inhibition of ovarian cancer by RGD-P125A-endostatin-Fc fusion proteins. Int. J. Cancer 129, 751–761. 10.1002/ijc.25932 21225621 PMC3156090

[B109] Kalin R. E. Kretz M. P. Meyer A. M. Kispert A. Heppner F. L. Brandli A. W. (2007). Paracrine and autocrine mechanisms of apelin signaling govern embryonic and tumor angiogenesis. Dev. Biol. 305, 599–614. 10.1016/j.ydbio.2007.03.004 17412318

[B110] Katoh M. (2016). Therapeutics targeting FGF signaling network in human diseases. Trends Pharmacol. Sci. 37, 1081–1096. 10.1016/j.tips.2016.10.003 27992319

[B111] Khalique S. Banerjee S. (2017). Nintedanib in ovarian cancer. Expert Opin. Investig. Drugs 26, 1073–1081. 10.1080/13543784.2017.1353599 28721753

[B112] Khan K. A. Kerbel R. S. (2018). Improving immunotherapy outcomes with anti-angiogenic treatments and vice versa. Nat. Rev. Clin. Oncol. 15, 310–324. 10.1038/nrclinonc.2018.9 29434333

[B113] Kim B. J. Kim D. Kim J. H. Kim H. S. Jang H. J. (2021). The efficacy and safety of onartuzumab in patients with solid cancers: A meta-analysis of randomized trials. Indian J. Cancer 58, 232–240. 10.4103/ijc.IJC_797_18 33402588

[B114] Kloepper J. Riedemann L. Amoozgar Z. Seano G. Susek K. Yu V. (2016). Ang-2/VEGF bispecific antibody reprograms macrophages and resident microglia to anti-tumor phenotype and prolongs glioblastoma survival. Proc. Natl. Acad. Sci. U. S. A. 113, 4476–4481. 10.1073/pnas.1525360113 27044098 PMC4843473

[B115] Krusche B. Ottone C. Clements M. P. Johnstone E. R. Goetsch K. Lieven H. (2016). EphrinB2 drives perivascular invasion and proliferation of glioblastoma stem-like cells. Elife 5, e14845. 10.7554/eLife.14845 27350048 PMC4924994

[B116] Lacquaniti A. Altavilla G. Picone A. Donato V. Chirico V. Mondello P. (2015). Apelin beyond kidney failure and hyponatremia: A useful biomarker for cancer disease progression evaluation. Clin. Exp. Med. 15, 97–105. 10.1007/s10238-014-0272-y 24469934

[B117] Lamalice L. Le Boeuf F. Huot J. (2007). Endothelial cell migration during angiogenesis. Circ. Res. 100, 782–794. 10.1161/01.RES.0000259593.07661.1e 17395884

[B118] Langenkamp E. Zhang L. Lugano R. Huang H. Elhassan T. E. Georganaki M. (2015). Elevated expression of the C-type lectin CD93 in the glioblastoma vasculature regulates cytoskeletal rearrangements that enhance vessel function and reduce host survival. Cancer Res. 75, 4504–4516. 10.1158/0008-5472.CAN-14-3636 26363010

[B119] Lassus H. Sihto H. Leminen A. Nordling S. Joensuu H. Nupponen N. N. (2004). Genetic alterations and protein expression of KIT and PDGFRA in serous ovarian carcinoma. Br. J. Cancer 91, 2048–2055. 10.1038/sj.bjc.6602252 15583695 PMC2409787

[B120] Ledermann J. A. Embleton A. C. Raja F. Perren T. J. Jayson G. C. Rustin G. J. S. (2016). Cediranib in patients with relapsed platinum-sensitive ovarian cancer (ICON6): A randomised, double-blind, placebo-controlled phase 3 trial. Lancet 387, 1066–1074. 10.1016/S0140-6736(15)01167-8 27025186

[B121] Ledermann J. A. Embleton-Thirsk A. C. Perren T. J. Jayson G. C. Rustin G. J. S. Kaye S. B. (2021). Cediranib in addition to chemotherapy for women with relapsed platinum-sensitive ovarian cancer (ICON6): Overall survival results of a phase III randomised trial. ESMO Open 6, 100043. 10.1016/j.esmoop.2020.100043 33610123 PMC7903311

[B122] Ledermann J. A. Hackshaw A. Kaye S. Jayson G. Gabra H. McNeish I. (2011). Randomized phase II placebo-controlled trial of maintenance therapy using the oral triple angiokinase inhibitor BIBF 1120 after chemotherapy for relapsed ovarian cancer. J. Clin. Oncol. 29, 3798–3804. 10.1200/JCO.2010.33.5208 21859991

[B123] Lee C. K. Lee M. E. Lee W. S. Kim J. M. Park K. H. Kim T. S. (2015). Dovitinib (TKI258), a multi-target angiokinase inhibitor, is effective regardless of KRAS or BRAF mutation status in colorectal cancer. Am. J. Cancer Res. 5, 72–86. 25628921 PMC4300687

[B124] Lee J. M. Annunziata C. M. Hays J. L. Cao L. Choyke P. Yu M. (2020). Phase II trial of bevacizumab and sorafenib in recurrent ovarian cancer patients with or without prior-bevacizumab treatment. Gynecol. Oncol. 159, 88–94. 10.1016/j.ygyno.2020.07.031 32747013 PMC7541580

[B125] Lee J. M. Sarosy G. A. Annunziata C. M. Azad N. Minasian L. Kotz H. (2010). Combination therapy: Intermittent sorafenib with bevacizumab yields activity and decreased toxicity. Br. J. Cancer 102, 495–499. 10.1038/sj.bjc.6605514 20051952 PMC2822947

[B126] Lengyel E. (2010). Ovarian cancer development and metastasis. Am. J. Pathol. 177, 1053–1064. 10.2353/ajpath.2010.100105 20651229 PMC2928939

[B127] Leone Roberti Maggiore U. Valenzano Menada M. Venturini P. L. Ferrero S. (2013). The potential of sunitinib as a therapy in ovarian cancer. Expert Opin. Investig. Drugs 22, 1671–1686. 10.1517/13543784.2013.841138 24070205

[B128] Levitzki A. (2004). PDGF receptor kinase inhibitors for the treatment of PDGF driven diseases. Cytokine Growth Factor Rev. 15, 229–235. 10.1016/j.cytogfr.2004.03.010 15207814

[B129] Lheureux S. Oaknin A. Garg S. Bruce J. P. Madariaga A. Dhani N. C. (2020). Evolve: A multicenter open-label single-arm clinical and translational phase II trial of cediranib plus olaparib for ovarian cancer after PARP inhibition progression. Clin. Cancer Res. 26, 4206–4215. 10.1158/1078-0432.CCR-19-4121 32444417

[B130] Li J. Zhi X. Sun Y. Chen M. Yao L. (2022). The PDGF family is associated with activated tumor stroma and poor prognosis in ovarian cancer. Dis. Markers 2022, 5940049. 10.1155/2022/5940049 36199822 PMC9529473

[B131] Lisle J. E. Mertens-Walker I. Rutkowski R. Herington A. C. Stephenson S. A. (2013). Eph receptors and their ligands: Promising molecular biomarkers and therapeutic targets in prostate cancer. Biochim. Biophys. Acta 1835, 243–257. 10.1016/j.bbcan.2013.01.003 23396052

[B132] Liu J. F. Barry W. T. Birrer M. Lee J. M. Buckanovich R. J. Fleming G. F. (2014). Combination cediranib and olaparib versus olaparib alone for women with recurrent platinum-sensitive ovarian cancer: A randomised phase 2 study. Lancet Oncol. 15, 1207–1214. 10.1016/S1470-2045(14)70391-2 25218906 PMC4294183

[B133] Liu J. F. Barry W. T. Birrer M. Lee J. M. Buckanovich R. J. Fleming G. F. (2019). Overall survival and updated progression-free survival outcomes in a randomized phase II study of combination cediranib and olaparib versus olaparib in relapsed platinum-sensitive ovarian cancer. Ann. Oncol. 30, 551–557. 10.1093/annonc/mdz018 30753272 PMC6503628

[B134] Liu J. F. Brady M. F. Matulonis U. A. Miller A. Kohn E. C. Swisher E. M. (2022). Olaparib with or without cediranib versus platinum-based chemotherapy in recurrent platinum-sensitive ovarian cancer (NRG-GY004): A randomized, open-label, phase III trial. J. Clin. Oncol. 40, 2138–2147. 10.1200/jco.21.02011 35290101 PMC9242406

[B135] Liu J. F. Gordon M. Veneris J. Braiteh F. Balmanoukian A. Eder J. P. (2019). Safety, clinical activity and biomarker assessments of atezolizumab from a Phase I study in advanced/recurrent ovarian and uterine cancers. Gynecol. Oncol. 154, 314–322. 10.1016/j.ygyno.2019.05.021 31204078

[B136] Liu J. F. Herold C. Gray K. P. Penson R. T. Horowitz N. Konstantinopoulos P. A. (2019). Assessment of combined nivolumab and bevacizumab in relapsed ovarian cancer: A phase 2 clinical trial. JAMA Oncol. 5, 1731–1738. 10.1001/jamaoncol.2019.3343 31600397 PMC6802049

[B137] Liu X. Xu Y. Jin Q. Wang W. Zhang S. Wang X. (2016). EphA8 is a prognostic marker for epithelial ovarian cancer. Oncotarget 7, 20801–20809. 10.18632/oncotarget.8018 26989075 PMC4991493

[B138] Liu Y. Luo Y. Cai M. Shen P. Li J. Chen H. (2021). Anti-angiogenic therapy in ovarian cancer: Current situation and prospects. Indian J. Med. Res. 154, 680–690. 10.4103/ijmr.IJMR_1160_19 35532586 PMC9210530

[B139] Lopes-Coelho F. Martins F. Pereira S. A. Serpa J. (2021). Anti-angiogenic therapy: Current challenges and future perspectives. Int. J. Mol. Sci. 22, 3765. 10.3390/ijms22073765 33916438 PMC8038573

[B140] Lorusso D. Marchetti C. Conte C. Giudice E. Bolomini G. Vertechy L. (2020). Bevacizumab as maintenance treatment in BRCA mutated patients with advanced ovarian cancer: A large, retrospective, multicenter case-control study. Gynecol. Oncol. 159, 95–100. 10.1016/j.ygyno.2020.07.022 32703631

[B141] Lu C. Shahzad M. M. Moreno-Smith M. Lin Y. G. Jennings N. B. Allen J. K. (2010). Targeting pericytes with a PDGF-B aptamer in human ovarian carcinoma models. Cancer Biol. Ther. 9, 176–182. 10.4161/cbt.9.3.10635 20009575 PMC3155813

[B142] Lu C. Thaker P. H. Lin Y. G. Spannuth W. Landen C. N. Merritt W. M. (2008). Impact of vessel maturation on antiangiogenic therapy in ovarian cancer. Am. J. Obstet. Gynecol. 198, 477–e9. ; discussion 477 e479-410. 10.1016/j.ajog.2007.12.028 PMC2346589 18395047

[B143] Lu K. V. Chang J. P. Parachoniak C. A. Pandika M. M. Aghi M. K. Meyronet D. (2012). VEGF inhibits tumor cell invasion and mesenchymal transition through a MET/VEGFR2 complex. Cancer Cell 22, 21–35. 10.1016/j.ccr.2012.05.037 22789536 PMC4068350

[B144] Madsen C. V. Steffensen K. D. Olsen D. A. Waldstrom M. Smerdel M. Adimi P. (2012). Serial measurements of serum PDGF-AA, PDGF-BB, FGF2, and VEGF in multiresistant ovarian cancer patients treated with bevacizumab. J. Ovarian Res. 5, 23. 10.1186/1757-2215-5-23 22989094 PMC3511256

[B145] Mansouri A. McGregor N. Dunn R. Dobbie S. Holmes J. Collins L. (2021). Randomised phase II trial of olaparib, chemotherapy or olaparib and cediranib in patients with platinum-resistant ovarian cancer (OCTOVA): A study protocol. BMJ Open 11, e041463. 10.1136/bmjopen-2020-041463 PMC7813404 33452192

[B146] Maroto P. Porta C. Capdevila J. Apolo A. B. Viteri S. Rodriguez-Antona C. (2022). Cabozantinib for the treatment of solid tumors: A systematic review. Ther. Adv. Med. Oncol. 14, 17588359221107112. 10.1177/17588359221107112 35847482 PMC9284205

[B147] Marth C. Vergote I. Scambia G. Oberaigner W. Clamp A. Berger R. (2017). ENGOT-ov-6/TRINOVA-2: Randomised, double-blind, phase 3 study of pegylated liposomal doxorubicin plus trebananib or placebo in women with recurrent partially platinum-sensitive or resistant ovarian cancer. Eur. J. Cancer 70, 111–121. 10.1016/j.ejca.2016.09.004 27914241

[B148] Martinez-Bosch N. Navarro P. (2020). Galectins in the tumor microenvironment: Focus on galectin-1. Adv. Exp. Med. Biol. 1259, 17–38. 10.1007/978-3-030-43093-1_2 32578169

[B149] Matei D. Graeber T. G. Baldwin R. L. Karlan B. Y. Rao J. Chang D. D. (2002). Gene expression in epithelial ovarian carcinoma. Oncogene 21, 6289–6298. 10.1038/sj.onc.1205785 12214269

[B150] Mellman I. Coukos G. Dranoff G. (2011). Cancer immunotherapy comes of age. Nature 480, 480–489. 10.1038/nature10673 22193102 PMC3967235

[B151] Merritt W. M. Nick A. M. Carroll A. R. Lu C. Matsuo K. Dumble M. (2010). Bridging the gap between cytotoxic and biologic therapy with metronomic topotecan and pazopanib in ovarian cancer. Mol. Cancer Ther. 9, 985–995. 10.1158/1535-7163.MCT-09-0967 20371710 PMC2852465

[B152] Mielczarek-Palacz A. Kondera-Anasz Z. Smycz-Kubanska M. Englisz A. Janusz A. Krolewska-Daszczynska P. (2022). The role of galectins-1, 3, 7, 8 and 9 as potential diagnostic and therapeutic markers in ovarian cancer (Review). Mol. Med. Rep. 25, 166. 10.3892/mmr.2022.12682 35293602 PMC8941520

[B153] Mitra A. K. Sawada K. Tiwari P. Mui K. Gwin K. Lengyel E. (2011). Ligand-independent activation of c-Met by fibronectin and α(5)β(1)-integrin regulates ovarian cancer invasion and metastasis. Oncogene 30, 1566–1576. 10.1038/onc.2010.532 21119598 PMC3069218

[B154] Monk B. J. Han E. Josephs-Cowan C. A. Pugmire G. Burger R. A. (2006). Salvage bevacizumab (rhuMAB VEGF)-based therapy after multiple prior cytotoxic regimens in advanced refractory epithelial ovarian cancer. Gynecol. Oncol. 102, 140–144. 10.1016/j.ygyno.2006.05.006 16790264

[B155] Monk B. J. Minion L. E. Coleman R. L. (2016). Anti-angiogenic agents in ovarian cancer: Past, present, and future. Ann. Oncol. 27 (1), i33–i39. 10.1093/annonc/mdw093 27141068 PMC6283356

[B156] Monk B. J. Poveda A. Vergote I. Raspagliesi F. Fujiwara K. Bae D. S. (2014). Anti-angiopoietin therapy with trebananib for recurrent ovarian cancer (TRINOVA-1): A randomised, multicentre, double-blind, placebo-controlled phase 3 trial. Lancet Oncol. 15, 799–808. 10.1016/S1470-2045(14)70244-X 24950985

[B157] Monk B. J. Poveda A. Vergote I. Raspagliesi F. Fujiwara K. Bae D. S. (2016). Final results of a phase 3 study of trebananib plus weekly paclitaxel in recurrent ovarian cancer (TRINOVA-1): Long-term survival, impact of ascites, and progression-free survival-2. Gynecol. Oncol. 143, 27–34. 10.1016/j.ygyno.2016.07.112 27546885

[B158] Moroney J. W. Powderly J. Lieu C. H. Bendell J. C. Eckhardt S. G. Chang C. W. (2020). Safety and clinical activity of atezolizumab plus bevacizumab in patients with ovarian cancer: A phase Ib study. Clin. Cancer Res. 26, 5631–5637. 10.1158/1078-0432.CCR-20-0477 32723836

[B159] Motz G. T. Coukos G. (2011). The parallel lives of angiogenesis and immunosuppression: Cancer and other tales. Nat. Rev. Immunol. 11, 702–711. 10.1038/nri3064 21941296

[B160] Nagy J. A. Dvorak H. F. (2012). Heterogeneity of the tumor vasculature: The need for new tumor blood vessel type-specific targets. Clin. Exp. Metastasis 29, 657–662. 10.1007/s10585-012-9500-6 22692562 PMC3484269

[B161] Ng C. S. Zhang Z. Lee S. I. Marques H. S. Burgers K. Su F. (2017). CT perfusion as an early biomarker of treatment efficacy in advanced ovarian cancer: An ACRIN and GOG study. Clin. Cancer Res. 23, 3684–3691. 10.1158/1078-0432.CCR-16-1859 28174234 PMC5720368

[B162] Nishio S. Matsumoto K. Takehara K. Kawamura N. Hasegawa K. Takeshima N. (2020). Pembrolizumab monotherapy in Japanese patients with advanced ovarian cancer: Subgroup analysis from the KEYNOTE-100. Cancer Sci. 111, 1324–1332. 10.1111/cas.14340 32012411 PMC7156846

[B163] Nixon A. Liu J. Xiong N. Hurwitz H. I. Lyu J. Liu Y. (2021). Blood-based biomarkers in patients with platinum-sensitive and resistant ovarian cancer treated with olaparib and cediranib: Results from the UM9825 trial. Gynecol. Oncol. 162, S99. 10.1016/s0090-8258(21)00831-3

[B164] Norden A. D. Schiff D. Ahluwalia M. S. Lesser G. J. Nayak L. Lee E. Q. (2015). Phase II trial of triple tyrosine kinase receptor inhibitor nintedanib in recurrent high-grade gliomas. J. Neurooncol. 121, 297–302. 10.1007/s11060-014-1631-y 25338318

[B165] Noren N. K. Lu M. Freeman A. L. Koolpe M. Pasquale E. B. (2004). Interplay between EphB4 on tumor cells and vascular ephrin-B2 regulates tumor growth. Proc. Natl. Acad. Sci. U. S. A. 101, 5583–5588. 10.1073/pnas.0401381101 15067119 PMC397426

[B166] Numnum T. M. Rocconi R. P. Whitworth J. Barnes M. N. (2006). The use of bevacizumab to palliate symptomatic ascites in patients with refractory ovarian carcinoma. Gynecol. Oncol. 102, 425–428. 10.1016/j.ygyno.2006.05.018 16797681

[B167] Ogawa K. Pasqualini R. Lindberg R. A. Kain R. Freeman A. L. Pasquale E. B. (2000). The ephrin-A1 ligand and its receptor, EphA2, are expressed during tumor neovascularization. Oncogene 19, 6043–6052. 10.1038/sj.onc.1204004 11146556

[B168] Ostman A. (2017). PDGF receptors in tumor stroma: Biological effects and associations with prognosis and response to treatment. Adv. Drug Deliv. Rev. 121, 117–123. 10.1016/j.addr.2017.09.022 28970051

[B169] Oyama T. Ran S. Ishida T. Nadaf S. Kerr L. Carbone D. P. (1998). Vascular endothelial growth factor affects dendritic cell maturation through the inhibition of nuclear factor-kappa B activation in hemopoietic progenitor cells. J. Immunol. 160, 1224–1232. 10.4049/jimmunol.160.3.1224 9570538

[B170] Papa A. Zaccarelli E. Caruso D. Vici P. Benedetti Panici P. Tomao F. (2016). Targeting angiogenesis in endometrial cancer - new agents for tailored treatments. Expert Opin. Investig. Drugs 25, 31–49. 10.1517/13543784.2016.1116517 26560489

[B171] Papadopoulos N. Lennartsson J. (2018). The PDGF/PDGFR pathway as a drug target. Mol. Asp. Med. 62, 75–88. 10.1016/j.mam.2017.11.007 29137923

[B172] Patch A. M. Christie E. L. Etemadmoghadam D. Garsed D. W. George J. Fereday S. (2015). Whole-genome characterization of chemoresistant ovarian cancer. Nature 521, 489–494. 10.1038/nature14410 26017449

[B173] Pennington K. P. Swisher E. M. (2012). Hereditary ovarian cancer: Beyond the usual suspects. Gynecol. Oncol. 124, 347–353. 10.1016/j.ygyno.2011.12.415 22264603

[B174] Perren T. J. Swart A. M. Pfisterer J. Ledermann J. A. Pujade-Lauraine E. Kristensen G. (2011). A phase 3 trial of bevacizumab in ovarian cancer. N. Engl. J. Med. 365, 2484–2496. 10.1056/nejmoa1103799 22204725

[B175] Peterson T. E. Kirkpatrick N. D. Huang Y. Farrar C. T. Marijt K. A. Kloepper J. (2016). Dual inhibition of Ang-2 and VEGF receptors normalizes tumor vasculature and prolongs survival in glioblastoma by altering macrophages. Proc. Natl. Acad. Sci. U. S. A. 113, 4470–4475. 10.1073/pnas.1525349113 27044097 PMC4843449

[B176] Pietras K. Pahler J. Bergers G. Hanahan D. (2008). Functions of paracrine PDGF signaling in the proangiogenic tumor stroma revealed by pharmacological targeting. PLoS Med. 5, e19. 10.1371/journal.pmed.0050019 18232728 PMC2214790

[B177] Pignata S. Lorusso D. Joly F. Gallo C. Colombo N. Sessa C. (2021). Carboplatin-based doublet plus bevacizumab beyond progression versus carboplatin-based doublet alone in patients with platinum-sensitive ovarian cancer: A randomised, phase 3 trial. Lancet Oncol. 22, 267–276. 10.1016/S1470-2045(20)30637-9 33539744

[B178] Plummer R. Madi A. Jeffels M. Richly H. Nokay B. Rubin S. (2013). A Phase I study of pazopanib in combination with gemcitabine in patients with advanced solid tumors. Cancer Chemother. Pharmacol. 71, 93–101. 10.1007/s00280-012-1982-z 23064954 PMC3535414

[B179] Polcher M. Eckhardt M. Coch C. Wolfgarten M. Kubler K. Hartmann G. (2010). Sorafenib in combination with carboplatin and paclitaxel as neoadjuvant chemotherapy in patients with advanced ovarian cancer. Pharmacol 66, 203–207. 10.1007/s00280-010-1276-2 20204367

[B180] Poluzzi C. Iozzo R. V. Schaefer L. (2016). Endostatin and endorepellin: A common route of action for similar angiostatic cancer avengers. Adv. Drug Deliv. Rev. 97, 156–173. 10.1016/j.addr.2015.10.012 26518982 PMC4753091

[B181] Poveda A. M. Selle F. Hilpert F. Reuss A. Savarese A. Vergote I. (2015). Bevacizumab combined with weekly paclitaxel, pegylated liposomal doxorubicin, or topotecan in platinum-resistant recurrent ovarian cancer: Analysis by chemotherapy cohort of the randomized phase III AURELIA trial. J. Clin. Oncol. 33, 3836–3838. 10.1200/JCO.2015.63.1408 26282651

[B182] Pranjol M. Z. I. Zinovkin D. A. Maskell A. R. T. Stephens L. J. Achinovich S. L. Los D. M. (2019). Cathepsin L-induced galectin-1 may act as a proangiogenic factor in the metastasis of high-grade serous carcinoma. J. Transl. Med. 17, 216. 10.1186/s12967-019-1963-7 31269957 PMC6610868

[B183] Presta M. Dell'Era P. Mitola S. Moroni E. Ronca R. Rusnati M. (2005). Fibroblast growth factor/fibroblast growth factor receptor system in angiogenesis. Cytokine Growth Factor Rev. 16, 159–178. 10.1016/j.cytogfr.2005.01.004 15863032

[B184] Previs R. A. Bevis K. S. Huh W. Tillmanns T. Perry L. Moore K. (2014). A prognostic nomogram to predict overall survival in women with recurrent ovarian cancer treated with bevacizumab and chemotherapy. Gynecol. Oncol. 132, 531–536. 10.1016/j.ygyno.2014.01.036 24472410

[B185] Pujade-Lauraine E. Fujiwara K. Ledermann J. A. Oza A. M. Kristeleit R. Ray-Coquard I. L. (2021). Avelumab alone or in combination with chemotherapy versus chemotherapy alone in platinum-resistant or platinum-refractory ovarian cancer (JAVELIN ovarian 200): An open-label, three-arm, randomised, phase 3 study. Lancet Oncol. 22, 1034–1046. 10.1016/s1470-2045(21)00216-3 34143970

[B186] Pujade-Lauraine E. Hilpert F. Weber B. Reuss A. Poveda A. Kristensen G. (2012). Aurelia: A randomized phase III trial evaluating bevacizumab (BEV) plus chemotherapy (CT) for platinum (PT)-resistant recurrent ovarian cancer. OC) 30, LBA5002. 10.1200/JCO.2013.51.4489 24637997

[B187] Pujade-Lauraine E. Hilpert F. Weber B. Reuss A. Poveda A. Kristensen G. (2014). Bevacizumab combined with chemotherapy for platinum-resistant recurrent ovarian cancer: The AURELIA open-label randomized phase III trial. J. Clin. Oncol. 32, 1302–1308. 10.1200/JCO.2013.51.4489 24637997

[B188] Ramasubbaiah R. Perkins S. M. Schilder J. Whalen C. Johnson C. S. Callahan M. (2011). Sorafenib in combination with weekly topotecan in recurrent ovarian cancer, a phase I/II study of the Hoosier Oncology Group. Gynecol. Oncol. 123, 499–504. 10.1016/j.ygyno.2011.08.033 21955480

[B189] Ray-Coquard I. Cibula D. Mirza M. R. Reuss A. Ricci C. Colombo N. (2020). Final results from GCIG/ENGOT/AGO-OVAR 12, a randomised placebo-controlled phase III trial of nintedanib combined with chemotherapy for newly diagnosed advanced ovarian cancer. Int. J. Cancer 146, 439–448. 10.1002/ijc.32606 31381147

[B190] Reinthaller A. (2016). Antiangiogenic therapies in ovarian cancer. Memo 9, 139–143. 10.1007/s12254-016-0282-4 27752291 PMC5045478

[B191] Reiss Y. Knedla A. Tal A. O. Schmidt M. H. H. Jugold M. Kiessling F. (2009). Switching of vascular phenotypes within a murine breast cancer model induced by angiopoietin-2. J. Pathol. 217, 571–580. 10.1002/path.2484 19116989

[B192] Ribeiro A. R. G. Salvadori M. M. de Brot L. Bovolin G. Mantoan H. Ilelis F. (2021). Retrospective analysis of the role of cyclin E1 overexpression as a predictive marker for the efficacy of bevacizumab in platinum-sensitive recurrent ovarian cancer. Ecancermedicalscience 15, 1262. 10.3332/ecancer.2021.1262 34567247 PMC8426016

[B193] Rigamonti N. Kadioglu E. Keklikoglou I. Wyser Rmili C. Leow C. C. De Palma M. (2014). Role of angiopoietin-2 in adaptive tumor resistance to VEGF signaling blockade. Cell Rep. 8, 696–706. 10.1016/j.celrep.2014.06.059 25088418

[B194] Rini B. I. Michaelson M. D. Rosenberg J. E. Bukowski R. M. Sosman J. A. Stadler W. M. (2008). Antitumor activity and biomarker analysis of sunitinib in patients with bevacizumab-refractory metastatic renal cell carcinoma. J. Clin. Oncol. 26, 3743–3748. 10.1200/JCO.2007.15.5416 18669461

[B195] Robelin P. Tod M. Colomban O. Lachuer J. Ray-Coquard I. Rauglaudre G. (2020). Comparative analysis of predictive values of the kinetics of 11 circulating miRNAs and of CA125 in ovarian cancer during first line treatment (a GINECO study). Gynecol. Oncol. 159, 256–263. 10.1016/j.ygyno.2020.07.021 32712155

[B196] Ronca R. Giacomini A. Rusnati M. Presta M. (2015). The potential of fibroblast growth factor/fibroblast growth factor receptor signaling as a therapeutic target in tumor angiogenesis. Expert Opin. Ther. Targets 19, 1361–1377. 10.1517/14728222.2015.1062475 26125971

[B197] Ruscito I. Gasparri M. L. Marchetti C. De Medici C. Bracchi C. Palaia I. (2016). Cediranib in ovarian cancer: State of the art and future perspectives. Tumour Biol. 37, 2833–2839. 10.1007/s13277-015-4781-4 26753963

[B198] Salgado R. Benoy I. Weytjens R. Van Bockstaele D. Van Marck E. Huget P. (2002). Arterio-venous gradients of IL-6, plasma and serum VEGF and D-dimers in human cancer. Br. J. Cancer 87, 1437–1444. 10.1038/sj.bjc.6600655 12454774 PMC2376277

[B199] Sallinen H. Heikura T. Koponen J. Kosma V. M. Heinonen S. Yla-Herttuala S. (2014). Serum angiopoietin-2 and soluble VEGFR-2 levels predict malignancy of ovarian neoplasm and poor prognosis in epithelial ovarian cancer. BMC Cancer 14, 696. 10.1186/1471-2407-14-696 25245329 PMC4179851

[B200] Sallinen H. Heikura T. Laidinen S. Kosma V. M. Heinonen S. Yla-Herttuala S. (2010). Preoperative angiopoietin-2 serum levels: A marker of malignant potential in ovarian neoplasms and poor prognosis in epithelial ovarian cancer. Int. J. Gynecol. Cancer 20, 1498–1505. 10.1111/IGC.0b013e3181f936e3 21119365

[B201] Sawada K. Radjabi A. R. Shinomiya N. Kistner E. Kenny H. Becker A. R. (2007). c-Met overexpression is a prognostic factor in ovarian cancer and an effective target for inhibition of peritoneal dissemination and invasion. Cancer Res. 67, 1670–1679. 10.1158/0008-5472.CAN-06-1147 17308108

[B202] Sawamiphak S. Seidel S. Essmann C. L. Wilkinson G. A. Pitulescu M. E. Acker T. (2010). Ephrin-B2 regulates VEGFR2 function in developmental and tumour angiogenesis. Nature 465, 487–491. 10.1038/nature08995 20445540

[B203] Scharpfenecker M. Fiedler U. Reiss Y. Augustin H. G. (2005). The Tie-2 ligand angiopoietin-2 destabilizes quiescent endothelium through an internal autocrine loop mechanism. J. Cell Sci. 118, 771–780. 10.1242/jcs.01653 15687104

[B204] Schwandt A. von Gruenigen V. E. Wenham R. M. Frasure H. Eaton S. Fusco N. (2014). Randomized phase II trial of sorafenib alone or in combination with carboplatin/paclitaxel in women with recurrent platinum sensitive epithelial ovarian, peritoneal, or fallopian tube cancer. Invest. New Drugs 32, 729–738. 10.1007/s10637-014-0078-5 24619298

[B205] Seaman S. Stevens J. Yang M. Y. Logsdon D. Graff-Cherry C. St Croix B. (2007). Genes that distinguish physiological and pathological angiogenesis. Cancer Cell 11, 539–554. 10.1016/j.ccr.2007.04.017 17560335 PMC2039723

[B206] Secord A. A. McCollum M. Davidson B. A. Broadwater G. Squatrito R. Havrilesky L. J. (2019). Phase II trial of nintedanib in patients with bevacizumab-resistant recurrent epithelial ovarian, tubal, and peritoneal cancer. Gynecol. Oncol. 153, 555–561. 10.1016/j.ygyno.2019.03.246 30929823

[B207] Sharma R. Valls P. O. Inglese M. Dubash S. Chen M. Gabra H. (2020). [(18)F]Fluciclatide PET as a biomarker of response to combination therapy of pazopanib and paclitaxel in platinum-resistant/refractory ovarian cancer. Eur. J. Nucl. Med. Mol. Imaging 47, 1239–1251. 10.1007/s00259-019-04532-z 31754793 PMC7101300

[B208] Shimada C. Xu R. Al-Alem L. Stasenko M. Spriggs D. R. Rueda B. R. (2020). Galectins and ovarian cancer. Cancers (Basel) 12 (6), 1421. 10.3390/cancers12061421a 32486344 PMC7352943

[B209] Shojaei F. Lee J. H. Simmons B. H. Wong A. Esparza C. O. Plumlee P. A. (2010). HGF/c-Met acts as an alternative angiogenic pathway in sunitinib-resistant tumors. Cancer Res. 70, 10090–10100. 10.1158/0008-5472.CAN-10-0489 20952508

[B210] Song Y. Fu Y. Xie Q. Zhu B. Wang J. Zhang B. (2020). Anti-angiogenic agents in combination with immune checkpoint inhibitors: A promising strategy for cancer treatment. Front. Immunol. 11, 1956. 10.3389/fimmu.2020.01956 32983126 PMC7477085

[B211] Sorli S. C. Le Gonidec S. Knibiehler B. Audigier Y. (2007). Apelin is a potent activator of tumour neoangiogenesis. Oncogene 26, 7692–7699. 10.1038/sj.onc.1210573 17563744

[B212] Sorli S. C. van den Berghe L. Masri B. Knibiehler B. Audigier Y. (2006). Therapeutic potential of interfering with apelin signalling. Drug Discov. Today 11, 1100–1106. 10.1016/j.drudis.2006.10.011 17129829

[B213] Sostelly A. Mercier F. (2019). Tumor size and overall survival in patients with platinum-resistant ovarian cancer treated with chemotherapy and bevacizumab. Clin. Med. Insights Oncol. 13, 1179554919852071. 10.1177/1179554919852071 31191068 PMC6540487

[B214] Spiliotaki M. Markomanolaki H. Mela M. Mavroudis D. Georgoulias V. Agelaki S. (2011). Targeting the insulin-like growth factor I receptor inhibits proliferation and VEGF production of non-small cell lung cancer cells and enhances paclitaxel-mediated anti-tumor effect. Lung Cancer 73, 158–165. 10.1016/j.lungcan.2010.11.010 21190751

[B215] Steele I. A. Edmondson R. J. Bulmer J. N. Bolger B. S. Leung H. Y. Davies B. R. (2001). Induction of FGF receptor 2-IIIb expression and response to its ligands in epithelial ovarian cancer. Oncogene 20, 5878–5887. 10.1038/sj.onc.1204755 11593393

[B216] Steffensen K. D. Madsen C. V. Andersen R. F. Waldstrom M. Adimi P. Jakobsen A. (2014). Prognostic importance of cell-free DNA in chemotherapy resistant ovarian cancer treated with bevacizumab. Eur. J. Cancer 50, 2611–2618. 10.1016/j.ejca.2014.06.022 25087181

[B217] Sun S. Dong H. Yan T. Li J. Liu B. Shao P. (2020). Role of TSP-1 as prognostic marker in various cancers: A systematic review and meta-analysis. BMC Med. Genet. 21, 139. 10.1186/s12881-020-01073-3 32600280 PMC7325168

[B218] Tait C. R. Jones P. F. (2004). Angiopoietins in tumours: The angiogenic switch. J. Pathol. 204, 1–10. 10.1002/path.1618 15307132

[B219] Takahashi T. Yamaguchi S. Chida K. Shibuya M. (2001). A single autophosphorylation site on KDR/Flk-1 is essential for VEGF-A-dependent activation of PLC-gamma and DNA synthesis in vascular endothelial cells. EMBO J. 20, 2768–2778. 10.1093/emboj/20.11.2768 11387210 PMC125481

[B220] Thurston G. Rudge J. S. Ioffe E. Zhou H. Ross L. Croll S. D. (2000). Angiopoietin-1 protects the adult vasculature against plasma leakage. Nat. Med. 6, 460–463. 10.1038/74725 10742156

[B221] Tolkach Y. Ellinger J. Kremer A. Esser L. Muller S. C. Stephan C. (2019). Apelin and apelin receptor expression in renal cell carcinoma. Br. J. Cancer 120, 633–639. 10.1038/s41416-019-0396-7 30783205 PMC6461937

[B222] Topalian S. L. Taube J. M. Anders R. A. Pardoll D. M. (2016). Mechanism-driven biomarkers to guide immune checkpoint blockade in cancer therapy. Nat. Rev. Cancer 16, 275–287. 10.1038/nrc.2016.36 27079802 PMC5381938

[B223] Troncoso M. F. Ferragut F. Bacigalupo M. L. Cardenas Delgado V. M. Nugnes L. G. Gentilini L. (2014). Galectin-8: A matricellular lectin with key roles in angiogenesis. Glycobiology 24, 907–914. 10.1093/glycob/cwu054 24939370

[B224] Tuppurainen L. Sallinen H. Karvonen A. Valkonen E. Laakso H. Liimatainen T. (2017). Combined gene therapy using AdsVEGFR2 and AdsTie2 with chemotherapy reduces the growth of human ovarian cancer and formation of ascites in mice. Int. J. Gynecol. Cancer 27, 879–886. 10.1097/IGC.0000000000000973 28498260

[B225] Turner N. Grose R. (2010). Fibroblast growth factor signalling: From development to cancer. Nat. Rev. Cancer 10, 116–129. 10.1038/nrc2780 20094046

[B226] Uhl C. Markel M. Broggini T. Nieminen M. Kremenetskaia I. Vajkoczy P. (2018). EphB4 mediates resistance to antiangiogenic therapy in experimental glioma. Angiogenesis 21, 873–881. 10.1007/s10456-018-9633-6 29987450 PMC6208883

[B227] van Hinsbergh V. W. Koolwijk P. (2008). Endothelial sprouting and angiogenesis: Matrix metalloproteinases in the lead. Cardiovasc. Res. 78, 203–212. 10.1093/cvr/cvm102 18079100

[B228] Varga A. Piha-Paul S. Ott P. A. Mehnert J. M. Berton-Rigaud D. Morosky A. (2019). Pembrolizumab in patients with programmed death ligand 1-positive advanced ovarian cancer: Analysis of KEYNOTE-028. Gynecol. Oncol. 152, 243–250. 10.1016/j.ygyno.2018.11.017 30522700

[B229] Vergote I. B. Smith D. C. Berger R. Kurzrock R. Vogelzang N. J. Sella A. (2017). A phase 2 randomised discontinuation trial of cabozantinib in patients with ovarian carcinoma. Eur. J. Cancer 83, 229–236. 10.1016/j.ejca.2017.06.018 28755607

[B230] Vergote I. du Bois A. Floquet A. Rau J. Kim J. W. Del Campo J. M. (2019). Overall survival results of AGO-OVAR16: A phase 3 study of maintenance pazopanib versus placebo in women who have not progressed after first-line chemotherapy for advanced ovarian cancer. Gynecol. Oncol. 155, 186–191. 10.1016/j.ygyno.2019.08.024 31519320

[B231] Vergote I. Scambia G. O'Malley D. M. Van Calster B. Park S. Y. Del Campo J. M. (2019). Trebananib or placebo plus carboplatin and paclitaxel as first-line treatment for advanced ovarian cancer (TRINOVA-3/ENGOT-ov2/GOG-3001): A randomised, double-blind, phase 3 trial. Lancet Oncol. 20, 862–876. 10.1016/S1470-2045(19)30178-0 31076365

[B232] Viallard C. Larrivee B. (2017). Tumor angiogenesis and vascular normalization: Alternative therapeutic targets. Angiogenesis 20, 409–426. 10.1007/s10456-017-9562-9 28660302

[B233] Wang J. Y. Sun T. Zhao X. L. Zhang S. W. Zhang D. F. Gu Q. (2008). Functional significance of VEGF-a in human ovarian carcinoma: Role in vasculogenic mimicry. Cancer Biol. Ther. 7, 758–766. 10.4161/cbt.7.5.5765 18376140

[B234] Wang Y. Nakayama M. Pitulescu M. E. Schmidt T. S. Bochenek M. L. Sakakibara A. (2010). Ephrin-B2 controls VEGF-induced angiogenesis and lymphangiogenesis. Nature 465, 483–486. 10.1038/nature09002 20445537

[B235] Wang Y. Thai T. Moore K. Ding K. McMeekin S. Liu H. (2016). Quantitative measurement of adiposity using CT images to predict the benefit of bevacizumab-based chemotherapy in epithelial ovarian cancer patients. Oncol. Lett. 12, 680–686. 10.3892/ol.2016.4648 27347200 PMC4907303

[B236] White F. C. Benehacene A. Scheele J. S. Kamps M. (1997). VEGF mRNA is stabilized by ras and tyrosine kinase oncogenes, as well as by UV radiation--evidence for divergent stabilization pathways. Growth factors. 14, 199–212. 10.3109/08977199709021520 9255609

[B237] Willett C. G. Duda D. G. di Tomaso E. Boucher Y. Ancukiewicz M. Sahani D. V. (2009). Efficacy, safety, and biomarkers of neoadjuvant bevacizumab, radiation therapy, and fluorouracil in rectal cancer: A multidisciplinary phase II study. J. Clin. Oncol. 27, 3020–3026. 10.1200/JCO.2008.21.1771 19470921 PMC2702234

[B238] Wu L. Chen L. Li L. (2017). Apelin/APJ system: A novel promising therapy target for pathological angiogenesis. Clin. Chim. Acta 466, 78–84. 10.1016/j.cca.2016.12.023 28025030

[B239] Xie Y. Su N. Yang J. Tan Q. Huang S. Jin M. (2020). FGF/FGFR signaling in health and disease. Signal Transduct. Target Ther. 5, 181. 10.1038/s41392-020-00222-7 32879300 PMC7468161

[B240] Yu P. Wilhelm K. Dubrac A. Tung J. K. Alves T. C. Fang J. S. (2017). FGF-dependent metabolic control of vascular development. Nature 545, 224–228. 10.1038/nature22322 28467822 PMC5427179

[B241] Yuan H. T. Khankin E. V. Karumanchi S. A. Parikh S. M. (2009). Angiopoietin 2 is a partial agonist/antagonist of Tie2 signaling in the endothelium. Mol. Cell. Biol. 29, 2011–2022. 10.1128/MCB.01472-08 19223473 PMC2663314

[B242] Zhang D. Huang J. Sun Y. Guo Q. (2019). Long-term progression-free survival of apatinib monotherapy for relapsed ovarian cancer: A case report and literature review. Onco Targets Ther. 12, 3635–3644. 10.2147/OTT.S198946 31190866 PMC6529614

[B243] Zhang L. Yang N. Park J. W. Katsaros D. Fracchioli S. Cao G. (2003). Tumor-derived vascular endothelial growth factor up-regulates angiopoietin-2 in host endothelium and destabilizes host vasculature, supporting angiogenesis in ovarian cancer. Cancer Res. 63, 3403–3412. 12810677

[B244] Zhang X. Lawler J. (2007). Thrombospondin-based antiangiogenic therapy. Microvasc. Res. 74, 90–99. 10.1016/j.mvr.2007.04.007 17559888 PMC2100421

[B245] Zhang Y. Wang H. Oliveira R. H. M. Zhao C. Popel A. S. (2022). Systems biology of angiogenesis signaling: Computational models and omics. WIREs Mech. Dis. 14, e1550. 10.1002/wsbm.1550 34970866 PMC9243197

[B246] Zhao C. Isenberg J. S. Popel A. S. (2018). Human expression patterns: Qualitative and quantitative analysis of thrombospondin-1 under physiological and pathological conditions. J. Cell. Mol. Med. 22, 2086–2097. 10.1111/jcmm.13565 29441713 PMC5867078

[B247] Zhao Y. Adjei A. A. (2015). Targeting angiogenesis in cancer therapy: Moving beyond vascular endothelial growth factor. Oncologist 20, 660–673. 10.1634/theoncologist.2014-0465 26001391 PMC4571783

[B248] Zhou Y. Wu C. Lu G. Hu Z. Chen Q. Du X. (2020). FGF/FGFR signaling pathway involved resistance in various cancer types. J. Cancer 11, 2000–2007. 10.7150/jca.40531 32127928 PMC7052940

