# Supplementary material for: Anti-angiogenic therapy in ovarian cancer: Current understandings and prospects of precision medicine
Source: Front Pharmacol. 2023 Mar 7;14:1147717. doi: 10.3389/fphar.2023.1147717 (PMC10027942; doi:10.3389/fphar.2023.1147717)
Supplement: Supplementary file 1 [file Table1.DOCX]

Supplementary Table. Predictive Biomarkers for Antiangiogenic Drugs Treatment Outcome in clinical trials in ovarian cancer.

| **Study** | **Region** | **Cancer Type** | **Patients** | **Protocol** | **Study Design** | **Sample Type** | **Detect Method** | **Biomarker Type** | **biomarker** | **PFS** | | | | **OS** | | | |
| --- | --- | --- | --- | --- | --- | --- | --- | --- | --- | --- | --- | --- | --- | --- | --- | --- | --- |
|  |  |  |  |  |  |  |  |  |  | **HR** | **95L** | **95U** | **Pvalue** | **HR** | **95L** | **95U** | **P-value** |
| Steﬀensen et al. 2014 | USA | CT-resistant EOC | 144 | BEV | Prospective | plasma | RT-qPCR | DNA | cfDNA | 1.980 | 1.290 | 3.040 | 0.002 | 1.660 | 1.090 | 2.520 | 0.020 |
| Gao et al. 2020 | China | OC | 62 | CT＋BEV | Retrospective | tissue | NGS | DNA | EGFR status | 6.390 | 2.250 | 18.130 | 0.001 |  |  |  |  |
|  |  |  |  |  |  |  |  |  | HER2 status | 3.580 | 1.270 | 10.080 | 0.016 |  |  |  |  |
|  |  |  |  |  |  |  |  |  | MYC status | 0.214 | 0.045 | 1.015 | 0.052 |  |  |  |  |
| Ribeiro et al. 2021 | Brazil | Platinum-sensitive OC | 124 | CT±BEV | Retrospective | tissue | Immunohistochemistry | Protein | CCNE1 | 2.250 | 1.100 | 4.600 | 0.026 |  |  |  |  |
| Fabbi et al. 2022 | Italy | OC | 309 | PC＋BEV | Prospective | tissue | Immunohistochemistry | Protein | ADAM17 | 1.160 | 0.860 | 1.560 | 0.329 | 1.360 | 0.860 | 2.130 | 0.186 |
| Califano et al. 2021 | Italy | OC | 336 | CT＋BEV | Prospective | tissue | Immunohistochemistry and qRT-PCR | Protein and RNA | MVD | 0.740 | 0.490 | 1.130 | 0.165 | 0.860 | 0.480 | 1.530 | 0.601 |
|  |  |  |  |  |  |  |  |  | SMA_MVD | 0.800 | 0.530 | 1.220 | 0.295 | 0.740 | 0.370 | 1.490 | 0.406 |
|  |  |  |  |  |  |  |  |  | Ratio | 0.890 | 0.680 | 1.170 | 0.404 | 0.860 | 0.570 | 1.300 | 0.484 |
|  |  |  |  |  |  |  |  |  | miR-484 | 0.710 | 0.530 | 0.960 | 0.023 | 0.590 | 0.390 | 0.890 | 0.012 |
|  |  |  |  |  |  |  |  |  | VEGFA | 1.320 | 0.990 | 1.750 | 0.056 | 0.850 | 0.550 | 1.310 | 0.451 |
|  |  |  |  |  |  |  |  |  | VEGFB | 0.690 | 0.520 | 0.920 | 0.011 | 0.700 | 0.460 | 1.060 | 0.089 |
|  |  |  |  |  |  |  |  |  | VGFR2 | 1.090 | 0.750 | 1.600 | 0.642 | 1.190 | 0.690 | 2.030 | 0.531 |
|  |  |  |  |  |  |  |  |  | HIF1-α | 1.060 | 0.790 | 1.400 | 0.709 | 1.240 | 0.810 | 1.900 | 0.332 |
| Boisen et al. 2016 | Denmark | CT-refractory advanced EOC | 140 | BEV | Prospective | Plasma | ELISA | Protein | CA-125 | 0.970 | 0.780 | 1.210 | 0.782 | 1.040 | 0.870 | 1.250 | 0.677 |
|  |  |  |  |  |  |  |  |  | VEGF | 0.910 | 0.610 | 1.360 | 0.647 | 1.210 | 0.860 | 1.720 | 0.278 |
|  |  |  |  |  |  |  |  |  | YKL-40 | 2.910 | 1.070 | 7.920 | 0.036 | 1.970 | 0.900 | 4.320 | 0.089 |
| Secord et al. 2020 | USA | EOC | 751 | PC＋BEV | Prospective | Plasma | ELISA | Protein | OPN | 1.480 | 1.280 | 1.700 | 0.000 | 1.590 | 1.370 | 1.840 | 0.000 |
|  |  |  |  |  |  |  |  |  | IL6 | 1.140 | 1.070 | 1.210 | 0.000 | 1.170 | 1.100 | 1.260 | 0.000 |
|  |  |  |  |  |  |  |  |  | Ang-2 | 1.130 | 1.000 | 1.270 | 9.055 | 1.190 | 1.040 | 1.360 | 0.012 |
|  |  |  |  |  |  |  |  |  | SDF-1 | 1.070 | 0.990 | 1.150 | 0.078 | 1.050 | 0.970 | 1.140 | 0.224 |
|  |  |  |  |  |  |  |  |  | IL6R | 0.890 | 0.710 | 1.120 | 0.322 | 0.820 | 0.650 | 1.050 | 0.115 |
|  |  |  |  |  |  |  |  |  | GP130 | 0.890 | 0.700 | 1.140 | 0.373 | 0.880 | 0.680 | 1.150 | 0.350 |
|  |  |  |  |  |  |  |  |  | VEGF-D | 0.970 | 0.820 | 1.140 | 0.675 | 1.070 | 0.900 | 1.280 | 0.425 |
| Backen et al. 2014 | UK | OC | 205 | CT±BEV | Prospective | Plasma | ELISA | Protein | Ang1 | 0.413 | 0.146 | 1.168 | 0.096 |  |  |  |  |
|  |  |  |  |  |  |  |  |  | Tie2 | 0.463 | 0.138 | 1.546 | 0.210 |  |  |  |  |
| Collinson et al. 2013 | UK | EOC | 121 | CT＋BEV | Prospective | Serum | ELISA | Protein | Mesothelin | 2.000 | 1.220 | 3.280 | 0.006 |  |  |  |  |
|  |  |  |  |  |  |  |  |  | FLT4 | 2.330 | 1.480 | 3.680 | 0.000 |  |  |  |  |
|  |  |  |  |  |  |  |  |  | AGP | 2.000 | 1.230 | 3.260 | 0.005 |  |  |  |  |
|  |  |  |  |  |  |  |  |  | CA-125 | 2.130 | 1.340 | 3.370 | 0.001 |  |  |  |  |
| Halvorsen et al. 2017 | Europe, Canada, Australia and New Zealand | OC | 207 | PC±BEV | Prospective | plasma | RT-qPCR | miRNA | miR-1274A | 0.850 | 0.700 | 1.020 | 0.085 |  |  |  |  |
|  |  |  |  |  |  |  |  |  | miR-200b | 0.790 | 0.680 | 0.940 | 0.006 |  |  |  |  |
|  |  |  |  |  |  |  |  |  | miR-200c | 4.330 | 1.960 | 9.580 | 0.000 |  |  |  |  |
|  |  |  |  |  |  |  |  |  | miR-141 | 0.910 | 0.810 | 1.030 | 0.153 |  |  |  |  |
| Slaughter et al. 2014 | USA | EOC | 21 | CT＋BEV | Retrospective | patients | CT scan | Image | BMI | 5.160 | 1.310 | 20.240 | 0.020 | 2.490 | 0.810 | 7.690 | 0.110 |
|  |  |  |  |  |  |  |  |  | SFA | 1.190 | 0.370 | 3.820 | 0.770 | 3.580 | 1.120 | 11.430 | 0.030 |
|  |  |  |  |  |  |  |  |  | VFA | 1.760 | 0.580 | 5.320 | 0.320 | 1.760 | 0.580 | 5.320 | 0.320 |
| Buechel et al. 2021 | USA | EOC | 1249 | PC±BEV | Prospective | patients | CT scan | Image | BMI |  |  |  |  | 1.020 | 0.950 | 2.080 | 0.650 |
|  |  |  |  |  |  |  |  |  | SFA |  |  |  |  | 0.980 | 0.920 | 1.050 | 0.570 |
|  |  |  |  |  |  |  |  |  | SFD |  |  |  |  | 1.120 | 1.050 | 1.190 | 0.001 |
|  |  |  |  |  |  |  |  |  | VFA |  |  |  |  | 1.000 | 0.940 | 1.070 | 0.930 |
|  |  |  |  |  |  |  |  |  | VFD |  |  |  |  | 1.130 | 1.050 | 1.200 | 0.000 |
| Ng et al. 2017 | USA | Advanced OC | 76 | PC±BEV | Prospective | patients | CT scan | Image | BF | 2.860 | 1.280 | 6.420 | 0.009 | 1.880 | 0.590 | 5.930 | 0.280 |
|  |  |  |  |  |  |  |  |  | BV | 1.610 | 0.680 | 3.820 | 0.280 | 1.710 | 0.460 | 6.290 | 0.420 |
|  |  |  |  |  |  |  |  |  | PS | 0.720 | 0.350 | 1.510 | 0.390 | 0.570 | 0.150 | 2.130 | 0.400 |
| Lorusso et al. 2020 | Italy | Advanced OC | 441 | PC±BEV | Retrospective | Clinical charts |  | Patient Characteristics | BRCA status | 0.600 | 0.450 | 0.810 | 0.001 | 0.340 | 0.190 | 0.610 | 0.000 |
|  |  |  |  |  |  |  |  |  | PDS/NACT | 0.690 | 0.510 | 0.920 | 0.010 | 0.520 | 0.320 | 0.860 | 0.010 |
|  |  |  |  |  |  |  |  |  | RT | 0.500 | 0.370 | 0.700 | 0.000 | 0.680 | 0.380 | 1.190 | 0.170 |
| Tate et al. 2021 | Japan | OCCC | 28 | PC±BEV | Retrospective | Clinical charts |  | Patient Characteristics. | PS | 0.280 | 0.090 | 0.940 | 0.049 | 0.330 | 0.100 | 1.030 | 0.058 |
|  |  |  |  |  |  |  |  |  | CR | 0.200 | 0.060 | 0.700 | 0.013 | 0.190 | 0.050 | 0.780 | 0.021 |
| Farolfi et al. 2018 | Italy | EOC | 74 | CT＋BEV | Retrospective | Blood | Blood test | Inflammatory Index | NLR | 0.830 | 0.470 | 1.470 | 0.522 | 0.860 | 0.380 | 1.930 | 0.708 |
|  |  |  |  |  |  |  |  |  | PLR | 0.990 | 0.530 | 1.860 | 0.985 | 0.770 | 0.340 | 1.760 | 0.534 |
|  |  |  |  |  |  |  |  |  | SII | 0.840 | 0.470 | 1.490 | 0.545 | 0.810 | 0.360 | 1.850 | 0.621 |
| Nixon et al. 2021 | USA | OC | 70 | olaparib+cediranib | Prospective | Plasma | ELISA | Protein | OPN | 1.500 | 1.000 | 2.200 | 0.039 |  |  |  |  |
|  |  |  |  |  |  |  |  |  | IL-6 | 1.200 | 1.000 | 1.500 | 0.019 |  |  |  |  |
|  |  |  |  |  |  |  |  |  | TIMP-1 | 2.800 | 1.200 | 6.600 | 0.020 |  |  |  |  |
|  |  |  |  |  |  |  |  |  | Ang-2 | 1.700 | 1.100 | 2.800 | 0.023 |  |  |  |  |
| Robelin et al. 2020 | France | OC | 119 | PC±nintedanib | Prospective | Plasma | qRT-PCR | miRNA | miR-15b-5p | 0.630 | 0.570 | 0.690 | 0.000 | 0.630 | 0.530 | 0.730 | 0.001 |
|  |  |  |  |  |  |  |  |  | miR-34a | 0.640 | 0.520 | 0.760 | 0.009 | 0.660 | 0.560 | 0.760 | 0.000 |
| Sharma et al. 2020 | UK | Platinum-resistant/refractory OC | 16 | P+pazopanib | Prospective | Patients | CT scan | Image | Baseline SUV_60,mean_ | 1.750 | 0.930 | 3.300 | 0.080 |  |  |  |  |
| Bauerschlag et al. 2013 | Germany | Platinum-resistant/refractory OC | 43 | sunitinib | Prospective | Serum | ELISA | Protein | VEGF | 1.001 | 1.000 | 1.002 | 0.211 |  |  |  |  |
|  |  |  |  |  |  |  |  |  | Ang-2 | 1.000 | 1.000 | 1.001 | 0.813 |  |  |  |  |
|  |  |  |  |  |  |  |  |  | sVEGFR-3 | 1.000 | 1.000 | 1.000 | 0.389 |  |  |  |  |

[Abbreviation](http://www.baidu.com/link?url=kbB6nCKW3HlL3hcYYEkn70MF4vNjQIH1cgV-QoodbWq8SGSl92fAS0Ubj6Yl2anToNzIgHTuHxzh-fruRJiXnqkCXqOgTeRApfQoe4qLeS_): PFS, progress free survival; OS, overall survival; HR, hazard ratio; 95U, upper of 95% confidence interval; 95L, lower of 95% confidence interval; NGS, Next Generation Sequencing; BEV: Bevacizumab; CTa: Chemotherapy; CTb: Computed Tomography; PC：Paclitaxel and Carboplatin；DNA, Deoxyribonucleic Acid; RNA, Ribonucleic Acid; cfDNA, cell-free DNA; EGFR, Epidermal Growth Factor Receptor; HER2, Human Epidermal GrowthFactor Receptor 2; MYC, MYC Proto-Oncogene; CCNE1, Cyclin E1; ADAM17, a disintegrin and metalloprotease 17; MVD, microvessel density; SMA_MVD: Alfa-Smooth Muscle Actin+microvessel density; Ratio, α-SMA+MVD/MVD ratio; miRNA, microRNA; VEGFA, vascular endothelial growth factor A; VEGFB, vascular endothelial growth factor B; HIF-a, Hypoxia-Inducible Factor 1-alpha; OPN, osteopontin; SDF-1, stromal cell–derived factor-1; IL6R, IL6 receptor; FLT4, fms-like tyrosine kinase-4; AGP, a 1 -acid glycoprotein; BMI, body mass index; VFA, visceral fat area; SFA, subcutaneous fat area; ΔBF, change of Tumor Blood Flow; ΔBV, change of Tumor Blood Volume; ΔPS, change of Vessel Permeability Surface Product; PDS/NACT, Primary Debulking Surgery/Neoadjuvant chemotherapy; RT, Residual Tumor; PS, Performance Status; CR, Completeness of resection; NLR, neutrophil-to-lymphocyte ratio; PLR, platelet-to-lymphocyte ratio; SII, systemic immune inflammation index; SFD, subcutaneous fat density; VFD, visceral fat density.
